# Pseudopeptidic Coordination Polymers Based on Zirconium-Carboxylate Supramolecular Assemblies

**DOI:** 10.1021/acsami.5c02827

**Published:** 2025-05-05

**Authors:** Miguel Maireles-Porcar, Ferran Esteve, Nuria Martín, Julián Sanchez-Velandia, Belén Altava, Francisco G. Cirujano, Eduardo García-Verdugo

**Affiliations:** † Departamento de Química Inorgánica y Orgánica, 16748 Universitat Jaume I, Av. Vicent Sos Baynat, s/n, 12006 Castelló de la Plana, Castelló, España; ‡ Laboratoire de Chimie Supramoléculaire, Institut de Science et d’Ingénierie Supramoléculaires (ISIS), Université de Strasbourg, 8 allée Gaspard Monge, 6700 Strasbourg, France

**Keywords:** metal−organic frameworks, pseudopeptide, coordination polymer, supramolecular self-assembly, catalysis

## Abstract

Mimicking enzymes with new materials is a promising approach to improve efficiency and sustainability in heterogeneous catalysis. In this contribution, a family of coordination polymers based on *N*, *N*′-bis­(amino acid)­pyromellitic diimide linkers and Zr-oxo clusters has been assembled under solvothermal conditions in the presence of different acids (acetic, hydrochloric, and formic acid). The linker has been prepared from widely available amino acids and pyromellitic anhydride under microwave conditions. Different characterization techniques, such as NMR, Fourier transform infrared spectroscopy (FTIR), TGA, X-ray photoelectron spectroscopy (XPS), and scanning electron microscopy (SEM)/EDX, confirmed the formation of the pseudopeptidic (PSP) linkers and the subsequent formation of Zr-carboxylate bonds in the Zr-PSP coordination polymer, forming regular homogeneous nanoparticles with hybrid inorganic–organic composition. The PSPs have also been incorporated into defective UiO-67 crystals and employed as catalysts in the hydrolysis of *p*-nitrophenylacetate under mild conditions, exhibiting a correlation between porosity, residue volume, and activity.

## Graphical abstract

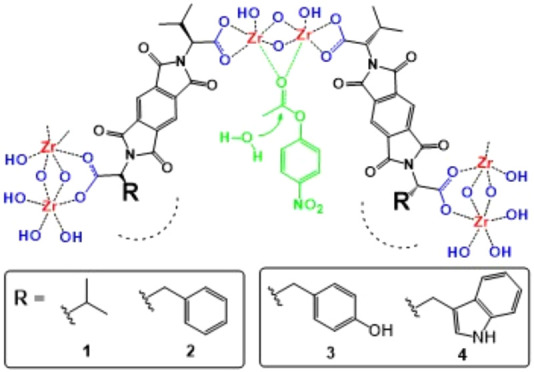

## Introduction

The reticular chemistry and the chemistry of coordination space developed during the last two decades have enabled the design and construction of new materials with two-dimensional (2D) or three-dimensional (3D)-nanostructured topologies and functionalities, the so-called Metal–Organic Frameworks (MOFs).^1^ These inorganic–organic hybrid polymers are based on coordination bonds between metal ions (connectors) and organic polytopic molecules (ligands) that lead to porous supramolecular architectures with preorganized active sites in confined chemical microenvironments,^2^ showcasing the catalytic portfolio found in some enzymes.^3,4^ Several groups have further exploited such enzyme-like behaviors through the postsynthetic incorporation of peptide-based scaffolds in the organic units of the MOF, commonly by the functionalization of amino groups present in aromatic linkers.^5–7^ Since many synthetic steps are required to incorporate small amounts of such peptidic sites into the final heterogeneous systems, the use of oligopeptides as linkers for the coordination polymer synthesis with divalent metal cations (*e.g*., Zn^2+^, Cu^2+^) has also been considered, resulting in peptide-based porous materials.^8–20^

An alternative to the use of peptides as linkers is the study of pseudopeptidic polytopic systems containing rigidized cores functionalized with amino acid units. For instance, pseudopeptides with aromatic diimides allowed for the construction of chiral coordination polymers upon binding to metal cations.^21^ The most employed metallic cores for the supramolecular coordination with the carboxylic acid units of amino acids are divalent cations such as Ca^2+^, Cd^2+^, Mn^2+^, Co^2+^, Zn^2+^, or Fe^2+^. Nevertheless, the resulting polymers present poor stability due to relatively weak coordination bonds between soft acids (M^2+^) and hard basic groups (COO^2–^). In contrast, the most stable MOFs reported to date present strong metal–oxygen–carbon bonds, through the coordination of Lewis acid metal cations to linear and planar aromatic dicarboxylates. In particular, the materials containing high charge/low size (hard) Lewis acid metals or secondary building units (SBU) (*e.g*., Zr-oxo clusters) coordinated to benzene-1,4-di- or 1,3,5-tricarboxylic acids are the most robust MOFs because of the high coordination number and stability of hard–hard Lewis acid–base Zr–O interactions.^22^ Some inspiring precedents have reported the postfunctionalization of the SBU units of Zr-carboxylate-based MOFs (*e.g*. UiO and MOF-808 type) with amino acid scaffolds, as well as the direct reaction of amino acids with the zirconium precursors.^23^ Although linear and planar aromatic diimide linkers with free aryl carboxylates have been exploited for the construction of Zr-MOFs,^24–27^ the use of the pseudopeptidic diimide analogs remains unexplored.

Therefore, we report herein a series of new pseudopeptidic coordination polymers based on Zr-carboxylate supramolecular assemblies. The pseudopeptidic ligands (namely **PSP-1–4**, Scheme 1) were prepared by reacting the enantiopure parent amino acids with pyromellitic anhydride under microwave conditions.^28^ These diimide pseudopeptides were then coordinated to the Zr^4+^ metallic centers in the presence of different acids, triggering the growth of 3D-nanostructured materials.

**Scheme 1:**
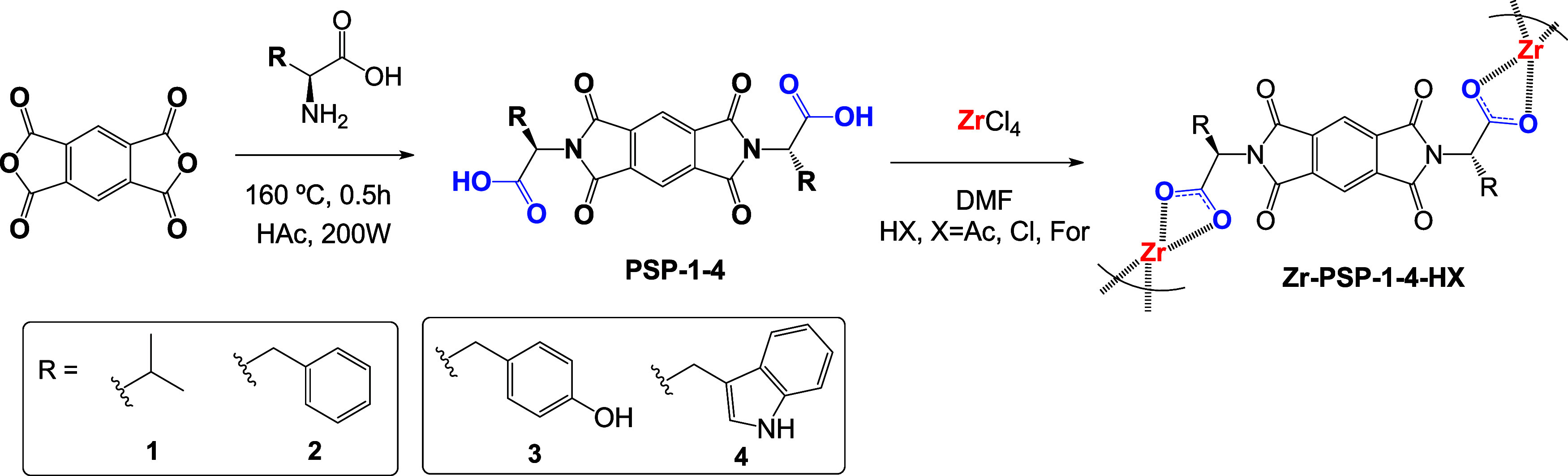
Synthesis of Pseudopeptidic **PSP** Linkers from Pyromellitic Dianhydride and Different Amino Acids (**1-4**) (First Step) and Supramolecular Assembly of **Zr-PSP** Coordination Polymers (Second Step)[Fn s1fn1]

a
**PSP-1** (valine), **PSP-2** (phenylalanine); **PSP-3** (tyrosine); **PSP-4** (tryptophan).

## Experimental Section

### Materials

The reagents used for the coordination polymers, pyromellitic dianhydride (97%), l-valine (99%), l-phenylalanine (>98%), l-tyrosine (>99%), l-tryptophan (99%), acetic acid (99%), formic acid (>95%), hydrochloric acid (37%), zirconium chloride (99%), *N*, *N*-dimethylformamide (99.8%), were supplied by Sigma-Aldrich-

### Structural Characterization

The porous texture of the samples was analyzed from the adsorption isotherms of N_2_ at 77 and 273 K. The equipment used was the automatic Micromeritics ASAP 2010 volumetric adsorption. The specific surface area was obtained from the Brunauer–Emmett–Teller (BET) equation and N_2_ adsorption isotherms. X-ray photoelectron spectroscopy (XPS) measurements were performed on a SPECS spectrometer equipped with a PHOIBOS 150 MCD–9 analyzer using a nonmonochromatic Mg KR (12536 eV) X-ray source working at 50 W. Microscopy images were obtained with a Hitachi S4800 (SEM-FEG) scanning electron microscopy- field emission gun equipment, with an accelerating voltage of 20 kV, coupled with an Energy Dispersive X-ray (EDX) detector. FT-IR spectra were acquired with a Pike single-reflection ATR diamond/ZnSe accessory in a JASCO FT/IR-4700 instrument. X-ray diffraction patterns were obtained with a D4 Endeavor, Bruker-AXS powder diffractometer. TGA- DSC3Mettler Toledo was coupled to a quadrupole mass spectrometer PFEIFFER VACUUM model OmniStar GSD 320 O3, 1–300 uma. ^1^H NMR was analyzed with a Bruker Avance III HD 300 MHz spectrometer.

### Synthesis of the Pseudopeptides

8.5 mmol of the amino acid and 4.5 mmol of pyromellitic dianhydride were weighed in a microwave tube, dissolved in 5 mL of acetic acid, and placed in the microwave oven at 200 W, 160 °C, 200 PSI for 0.5 h. The resulting pseudopeptide was precipitated after the addition of water. The solid was filtered and washed by redissolving in THF and was evaporated under vacuum.

### Synthesis of Zr-PSP Coordination Polymers

0.23 mmol portion of ZrCl_4_ and 0.23 mmol of PSP-1–4 were dissolved in 9 mL of DMF and 1 mL of formic acid (HFor). The solution was placed in an oven at 120 °C for 1 day, and the powder was isolated by centrifugation, washed with methanol, and dried overnight (see Supporting Information for details).

### Synthesis of PSP-Zr-BPDC Coordination Polymers

105 mg portion of ZrCl_4_ and 23 mg of BPDC (20% mol with respect to Zr) were dissolved in 18 mL of DMF and 2 mL of HFor. The suspension was heated in an oven at 120 °C for 24 h. Then, 1 eq. of PSP was added and left for another 24 h. This operation was repeated twice. The solid was isolated by filtration and washed 3 times with DMF to remove the PSP that is not incorporated into the solution.

## Results and Discussion

### Synthesis and Characterization of Zr-PSP-1

Pseudopeptidic ligands were synthesized according to previously described protocols, using microwaves as a green methodology.^28^ One may realize that the use of microwaves instead of conventional heating results in a cleaner and faster linker synthesis and requires less solvent-intensive isolation and purification steps.^29,30^ The main advantages of such ligands are their low cost, low toxicity, and structural tunability and functional density conferred by the chiral amino acid side chains. The solvothermal preparation of the pseudopeptidic coordination polymer **Zr-PSP-1** relied on the reaction between equimolar amounts of Zr^4+^ (as its chloride salt) and the chiral ditopic **PSP-1**, using low volumes of DMF to ensure appropriate interaction between precursors and solvent (see Scheme 1). According to positive effects when using acids as modulators of the Zr-MOFs crystal growth,^31^ different organic (*i.e*., acetic/formic acid) and inorganic (*i.e*., hydrochloric acid) additives were assayed, as indicated in Scheme 1 (see HX, where X = acetate, formate, and chloride). The relatively dynamic structure of **PSP-1** generated some disorder within the 3D-nanostructure of the materials, as shown by the broad signals assigned to amorphous polymers in the powder X-ray diffraction (XRD) analyses of **Zr-PSP-1-HAc**, **Zr-PSP-1-HCl**, and **Zr-PSP-1-HFor** (see Figure S5).

Consequently, the structural composition was studied using other techniques, such as Fourier transform infrared spectroscopy (FTIR), TGA, XPS, and SEM analysis. The redshift for the in-phase COO stretching modes in the FTIR spectra of **Zr-PSP-1**, in comparison to that of **PSP-1** (Figure 1a), indicated the formation of Zr-carboxylate bonds (≈ 1580 and 1707 cm^–1^, respectively). Moreover, the out-of-phase COO stretching of the carboxylic acid groups in **PSP-1** (intense doublet at 1335 and 1380 cm^–1^) slightly changed into a single band centered at 1365 cm^–1^ for **Zr-PSP-1**, supporting the formation of Zr-carboxylate coordination bonds. The band at 1655 cm^–1^ present in all **Zr-PSP-1** is likely the result of some DMF occluded in the structure (see Figure S6), while the band at 1715–1720 cm^–1^ in the **Zr-PSP-1** samples might correspond to noncoordinated carboxylic acid groups. The ratio of the Zr-coordinated (1560–1590 cm^–1^) to noncoordinated (centered at 1707 cm^–1^) bands increased as follows: **Zr-PSP-1-HAc** (0.67) < **Zr-PSP-1-HCl** (0.74) < **Zr-PSP-1-HFor** (0.78) (see Table S1). The Zr–O–H vibrations at lower wavenumbers overlapped with those of the aromatic scaffold of the **PSP** linkers.^32^ The presence of intercalated linker within the **Zr-PSP-1** structures was ascribed to the poor solubility of **PSP-1** in traditional organic solvents employed for washing the coordination polymers (*e.g*., ethanol) and its propensity to self-assemble by means of strong supramolecular π-π interactions.^28^

**Figure 1:**
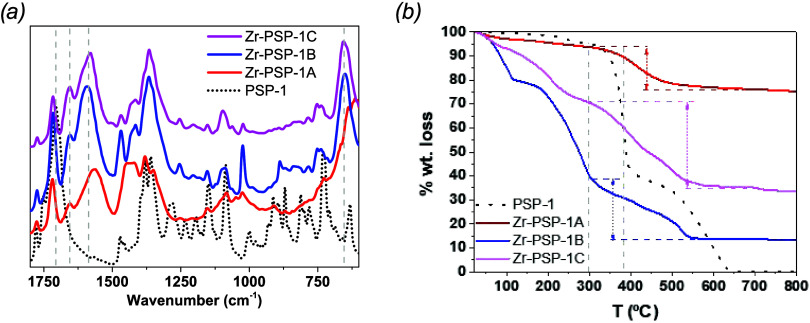
FTIR spectra (a) and TGA (b) of the pseudopeptidic linker **PSP-1** (black dots), and **Zr-PSP-1** coordination polymer prepared in the presence of acetic (red line), hydrochloric (blue line), and formic acid (magenta line).

Thermogravimetric analysis (TGA) of the samples corroborated the inorganic–organic hybrid nature of the coordination polymers, as an inorganic residue remained at *T* > 550 °C for all **Zr-PSP-1** (Figure 1b). The weight loss occurring at temperatures lower than 300 °C was attributed to small guest molecules (*i.e*., DMF, water, and acid modulators). This weight loss associated with guests (weakly bonded to the polymeric structure) was higher in the coordination polymer prepared using HCl (61% for **Zr-PSP-1-HCl**) than in the one synthesized using HFor (30% for **Zr-PSP-1-HFor**) or HAc (13% for **Zr-PSP-1-HAc**), as shown in Table S2. One may realize that the analogous analysis for **PSP** showed complete decomposition at such temperatures. Since the linker decomposed at temperatures >300 °C, we assumed the weight loss starting at this temperature and up to 600 °C as the amount of **PSP** present in the coordination polymer. The 300–600 °C weight loss increased in the order **Zr-PSP-1-HAc** (12%) < **Zr-PSP-1-HCl** (26%) < **Zr-PSP-1-HFor** (36%), in agreement with the degree of incorporation determined by FTIR (*vide supra*). The amount of Zr^4+^ incorporated in the coordination polymers followed the order: **Zr-PSP-1-HAc** (75%) > **Zr-PSP-1-HCl** (34%) > **Zr-PSP-1-HFor** (13%). When both the organic (**PSP-1**) and inorganic (ZrO_2_) components of the coordination polymers are expressed in moles, the following compositions (not considering the guest molecules) for the **Zr-PSP-1** coordination polymers can be obtained: (**HAc**) PSP_0.05_(ZrO_2_)_0.95_; (**HCl**) PSP_0.37_(ZrO_2_)_0.63_; and (**HFor**) PSP_0.24_(ZrO_2_)_0.76_. Therefore, these results confirm the poor incorporation of **PSP-1** linkers into the structure of the coordination polymer in the presence of acetic acid (material **Zr-PSP-1-HAc**).

X-photoelectron spectroscopy (XPS) analysis of the coordination polymer surface indicated the presence of both the inorganic (Zr) and organic (C, N, and O) building blocks in all coordination polymers, regardless of the acid modulator employed in their preparation (see Figure S7). Table S3 shows that while the proportion of C and O correlates with that of the parent **PSP-1**, the amount of N is slightly lower in all samples (especially for **Zr-PSP-1-HAc**). This might be due to inhomogeneities in the surface (but also to the presence of HAc in the structure, accounting for the higher amount of C and O observed). A similar scenario was found for the **PSP-1** linker, with the experimental composition of C_7.6_N_1_O_3.2_ being slightly different from the expected chemical formula (C_10_N_1_O_4_H_9_). The high-resolution XPS spectrum (O 1s) showed the bands at <531 eV indicative of Zr–O–Zr and Zr–O–C binding motifs of the MOFs, the bands associated with the imide (N–C=O) and ester (O–C=O) bonds between 531 and 534 eV, as well as bands at >534 eV suggesting the presence of O–H from noncoordinated carboxylic acid groups (see left part of Figure 2 and Tables S3–S4).^33–35^

**Figure 2:**
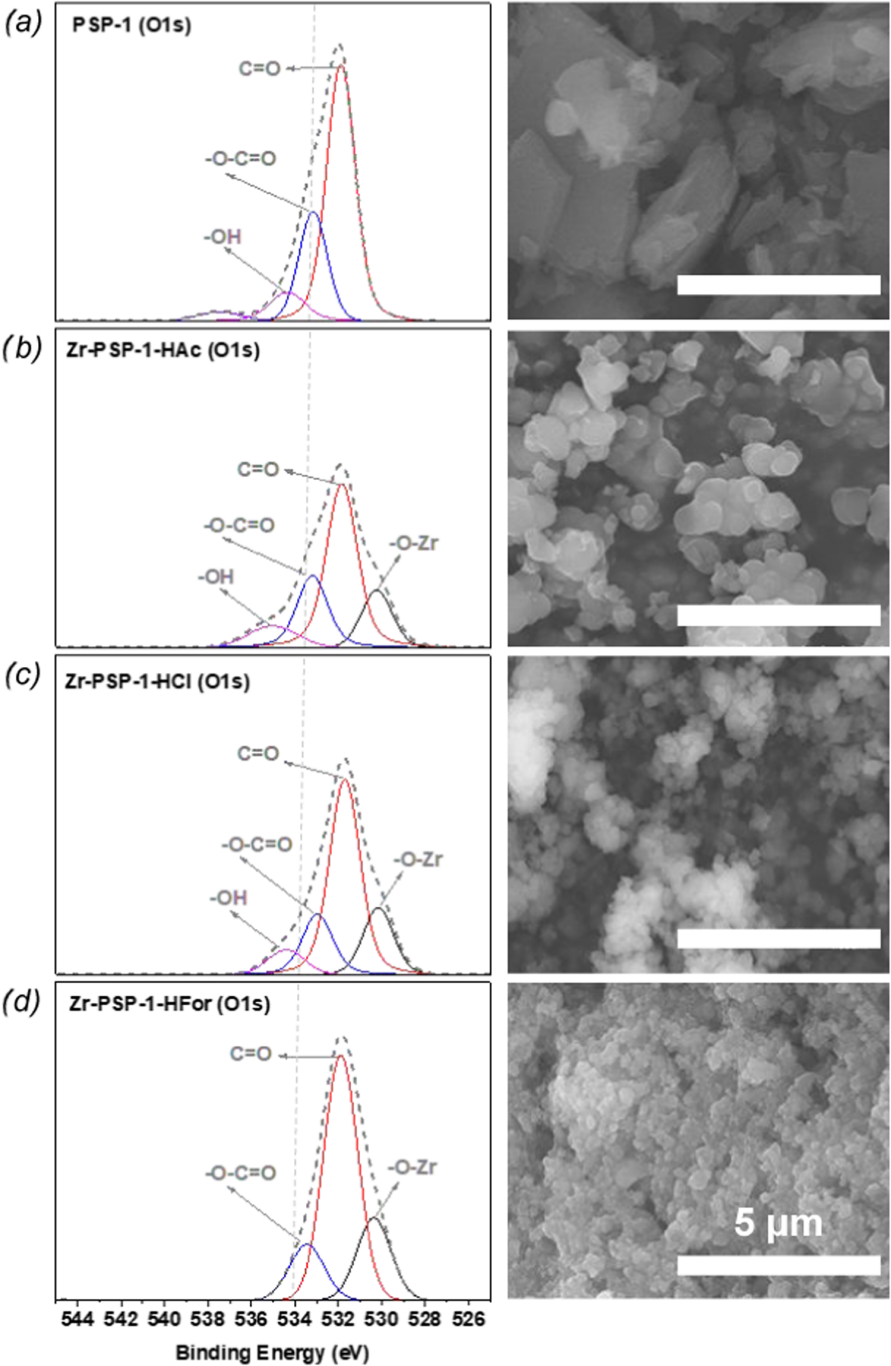
O 1s XPS spectra (left) and SEM images (right) of the pseudopeptidic linker PSP-1 (a), and **Zr-PSP-1** coordination polymer prepared in the presence of acetic (b), hydrochloric (c), and formic acid (d). Scale bar is 5 μm for all samples.

The Zr–O signal (*ca*. 530 eV) indicated the formation of Zr-carboxylate supramolecular assemblies, which area increased in the order: **Zr-PSP-1-HAc** (16.2%)< **Zr-PSP-1-HCl** (18.2%)< **Zr-PSP-1-HFor** (21.3%). The amount of free −COOH groups from **PSP-1** decreased in the order **Zr-PSP-1-HAc** (9.9%) > **Zr-PSP-1-HCl** (8.1%) > **Zr-PSP-1-HFor** (0%), in line with the Zr­(IV) coordination results above. Thus, both XPS and TGA outcomes corroborated the FTIR results, validating the use of this latter technique as a routine analysis for the assessment of such metal–organic structures. The higher proportion of Zr-carboxylate bonds in the formic acid-modulated coordination polymer (**Zr-PSP-1-HFor**), concerning that of **Zr-PSP-1-Hac**, could be a result of the lower p*K*_a_ of formic acid in comparison to that of acetic acid.^36^ SEM analyses were carried out to evaluate differences in morphology and size for the linker and coordination polymers formed (see the right part of Figure 2). The morphology of the **PSP-1** sample consisted of irregularly shaped microcrystals due to its layered intermolecular stacking *via* H-bonding and π-π stacking.^10^ In contrast, the **Zr-PSP-1** coordination polymers were assembled as homogeneous nanoparticles with regular shapes. It must be mentioned that the amount of free COOH groups found in the samples was proportional to the particle size of the coordination polymers: **Zr-PSP-1-HAc** > **Zr-PSP-1-HCl** > **Zr-PSP-1-HFor** (see left and right part of Figure 2). One notes that the formation of Zr–O coordination bonds with the carboxylic groups of **PSP-1** should preclude the intermolecular O–H bonds and π-π stacking of the aromatic diimide moiety, projecting the resulting coordination polymer in the three dimensions of space. It is possible that the intercalation and bonding of Zr^4+^ cations within the **PSP-1** layered structure (large micrometric scales) play a role in the (partial) destruction of the supramolecular 2D-layered structure and the formation of smaller (but probably multidimensional) 2*D*/3D (reticular)-Zr-PSP nanoparticles (see Scheme 2).

**Scheme 2:**
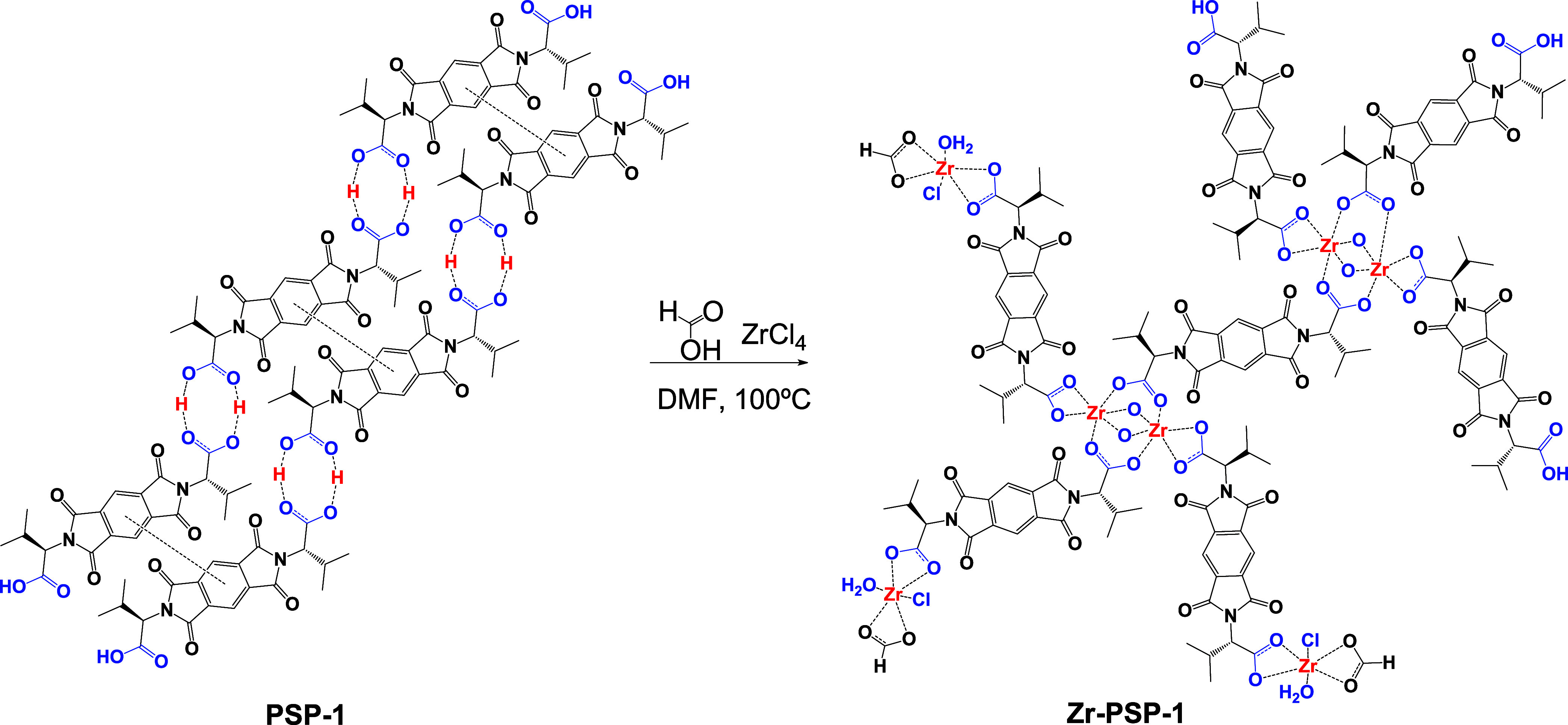
Tentative Structure of Zr-Carboxylate Coordination Polymers Generated from 2D-Layered PSPs, Formed upon H-Bonding and π-π Stacking, to 3D-Reticular Zr-PSPs, Formed by Coordination Bonds between Zr-Chloride/Formate and the Free Carboxylate Groups of PSP

### Synthesis and Characterization of Zr-PSPs with Different Amino Acids

To evaluate the effect of the amino acid side chain in the self-assembly of the materials, we synthesized three additional pseudopeptidic linkers based on phenylalanine, tyrosine, and tryptophan (*viz*. **PSP-2**, **PSP-3** and **PSP-4**, respectively). All of these pseudopeptides were obtained following a synthetic protocol similar to that of **PSP-1** (see SI for details). Given the encouraging results obtained when using **HFor** as an acid modulator, all of the syntheses of the coordination polymers derived from **PSP-2–4** were also carried out with this acid. This is the first time (to the best of our knowledge) that polar amino acids such as 3 and 4 (see Scheme 1) are employed in the pyromellitic diimide scaffold as linkers for the synthesis of coordination polymers, besides traditional nonpolar amino acids (*e.g*., 1 and 2 in Scheme 1). Linkers **PSP-2**, **PSP-3**, and **PSP-4** were synthesized in microwave conditions using acetic acid as the solvent, as described for **PSP-1**. The preparation of the pseudopeptidic **Zr-PSP-2**, **Zr-PSP-3**, and **Zr-PSP-4** coordination polymers was done using a similar solvothermal reaction (to that employed with **PSP-1**) between equimolar amounts of ZrCl_4_ and either PSP-2, PSP-3, or PSP-4, in the presence of DMF (see Scheme 1). In a similar manner to **Zr-PSP-1**, the resulting **Zr-PSP-2–4** were amorphous, as evidenced by the broad signals observed in the XRD patterns (Figure 3a).

**Figure 3:**
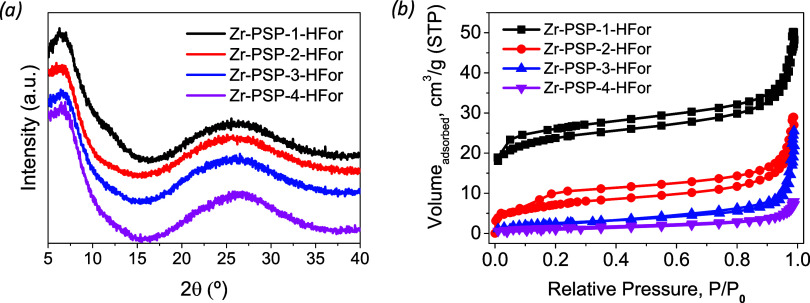
XRD patterns (a) and N_2_ physisorption isotherms (b) of the four Zr-PSPs prepared with different amino acids. PSP-1 (valine), PSP-2 (phenylalanine); PSP-3 (tyrosine); and PSP-4 (tryptophan).

We then evaluated the porosity of the hybrid materials. The N_2_ physisorption isotherms revealed slightly porous structures (Figure 3b), with **Zr-PSP-1** presenting the highest porosity. All samples present a hysteresis (H3–H4) curve between partial pressures of 0.2 and 0.8, indicating the presence of mesoporosity in the samples and typical groove pores generated by flaky particles. Interestingly, the porosity of the coordination polymers could be modulated through the side chain of the amino acid. Whereas the use of **PSP-4** barely provided porosity (surface area of 5 m^2^·g^–1^), the coordination polymer derived from **PSP-1** presented a surface area of *ca*. 80 m^2^·g^–1^ (See Table S5). This 15-fold increase in porosity was assigned to the less efficient supramolecular packing of **Zr-PSP-1** due to the sterically demanding isopropyl groups of the valine residue, which resulted in a higher number of defects in the 3D arrangement. On the other hand, the aromatic side chains of **PSP-2–4** were likely “closing” the structure through π-π attractive interactions that acted as additional cross-linking sites. We cannot neglect the effect of occluded **PSP** in the pores of the **Zr-PSP** materials, especially for **PSP-2–4** which are less soluble in DMF.

The FTIR spectra of the four coordination polymers revealed the presence of occluded DMF in the structure (Figure S8). The formation of Zr-carboxylate bonds was evident by the aforementioned redshift of the bands corresponding to the in-phase (*ca.* 1580 cm^–1^) and out-of-phase (*ca*. 1360 cm^–1^) COO stretching modes of **Zr-PSP-1–4**. The percentage of Zr-carboxylate bonds was quite high and similar in all samples (see Tables S3–S4): **Zr-PSP-1** (78%) ∼**Zr-PSP-2** (82%) ∼**Zr-PSP-3** (84%) ∼**Zr-PSP-4** (82%). These results stressed the importance of the acid modulator to facilitate network growth through dynamic ligand exchanges. The SEM images of the different **Zr-PSP-1–4** coordination polymers indicated the presence of nanoparticles of regular size and shape (Figure 4).

**Figure 4:**
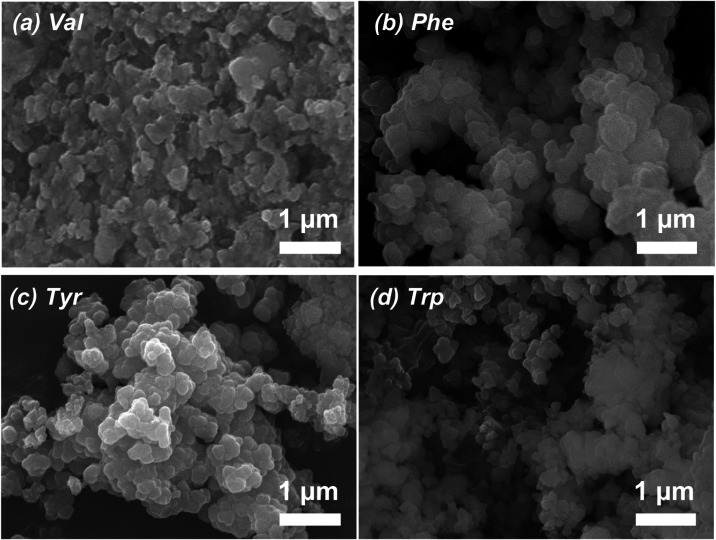
SEM images of the **Zr-PSP-1** (a), **Zr-PSP-2** (b), **Zr-PSP-3** (c), and **Zr-PSP-4** (d) coordination polymers prepared with different amino acids (see 1–4 in Scheme 2) in the presence of formic acid.

TGA results of the different **Zr-PSPs** samples (Figure 5) indicated a hybrid organic (34–37 wt %) and inorganic (34–44 wt %) composition, with about 21–30 wt % of guest molecules (removed at *T* < 300 °C), as indicated in Table S2. It can be inferred that the low volume of valine **1** allows for a higher amount of guest molecules (30 wt %) with respect to bulkier amino acids **2–4** (20 wt %), according to TGA (see weight loss below 200 °C in Figure 5 and Table S2).

**Figure 5:**
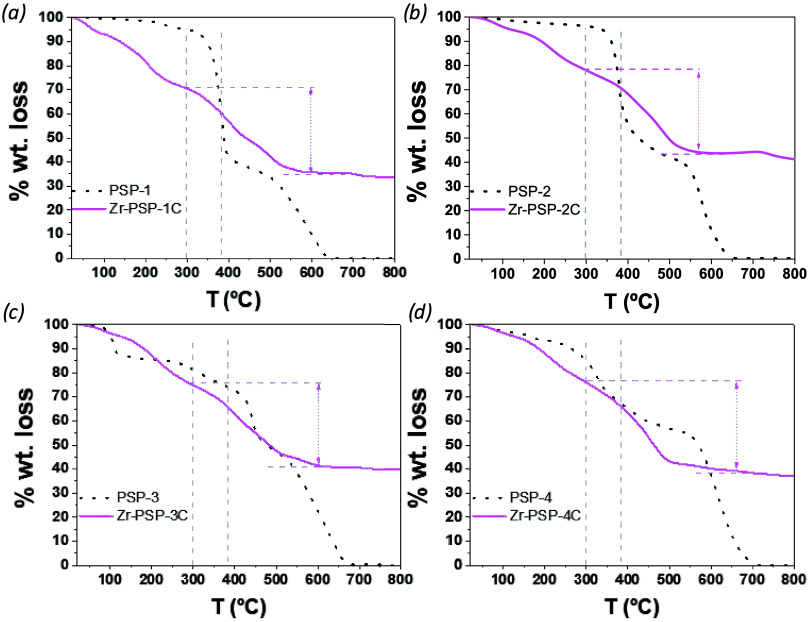
TGA of **Zr-PSP-1** (a), **Zr-PSP-2** (b), **Zr-PSP-3** (c), and **Zr-PSP-4** (d), all prepared with formic acid, with respect to the parent PSPs-1–4 linkers.

### Synthesis and Characterization of PSP-1–4-Zr-BPDC by PSP Incorporation into a Preformed Zr-BPDC with Linker Deficiency

We then decided to study the incorporation of the pseudopeptidic units into the UiO-67 framework to increase the crystallinity, porosity, and functionality of the resulting coordination polymers (*viz*. **Zr-PSP/BPDC** in Scheme 3). Taking into account the relatively similar size/composition of **PSP-1** and biphenyl-4,4′-dicarboxylic acid (**BPDC**), a synthetic approach based on the solvent-assisted linker exchange (SALE) of **BPDC** (at the preformed Zr-BPDC structure) by **PSP** linkers under solvothermal conditions was attempted.^37^ In particular, we assayed the formation of the heteroleptic polymers through a two-step protocol: initial synthesis of the **Zr-BPDC** coordination polymer with linker deficiency due to a low **BPDC**/Zr ratio (Scheme 3) followed by the addition of 3 equiv of **PSP-1–4** at different times (see SI for details).

**Scheme 3:**
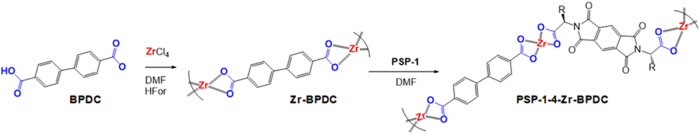
Supramolecular Assembly of PSP-Zr-BPDC Coordination Polymers *via* Solvent-Assisted Linker Exchange (SALE) of BPDC by PSP

The FTIR spectra for the different multicomponent materials revealed the presence of new signals, in addition to those of **Zr-BPDC** (*e.g*., peaks at 1590 and 1400 cm^–1^ in Figure 6) assigned to the partial incorporation of **PSP-1–4** (Figure 6a). For instance, all of the samples treated with the pseudopeptidic linker presented the **PSP**-**1–4** characteristic band centered at 1715 cm^–1^. The band observed at 1655 cm^–1^ was ascribed to the occluded DMF in all samples. One notes that the two-step SALE led to higher amounts of the pseudopeptide within the coordination polymer, as evidenced by the more intense band at 1715 cm^–1^ (also the one at 1375 cm^–1^). The spectra for these samples not only inferred the presence of pseudopeptidic linkers (bands at 1710–1720 cm^–1^) but also revealed a significant change in the structure of the **Zr-BPDC** backbone by means of a noteworthy shift in the C=O vibration of the metal carboxylates with respect to that of the pristine **Zr-BPDC**. In particular, the band at 1410 cm^–1^ ascribed to **Zr-BPDC** split into two peaks at 1415 cm^–1^ (minor intensity) and 1370 cm^–1^ (major intensity).

**Figure 6:**
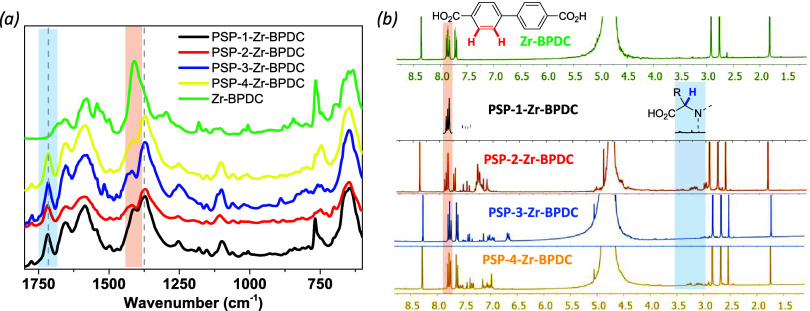
FTIR (a) and ^1^H NMR (b) spectra of the PSP-1-to-4-Zr-BPDC samples. The ^1^H NMR analysis was performed on the NH_4_HCO_3_ (1M)/ D_2_O digested sample (see Figure S10).

The organic composition of the hybrid material was determined by TGA (see Figure S11 and Table S6), and ^1^H NMR spectra of the four dissolved samples (Figure 6b), indicating the presence of both biphenyl (aromatic signals at 7.7–7.9 ppm) and **PSP** (aromatic signals of the pyromellitic scaffold at 7–7.7 ppm and the proton of the chiral site of the amino acids at *ca.* 3–4 ppm). Based on the proportion of these two signals, the amount of PSP incorporated into the PSP-Zr-BPDC coordination polymer increases in the order: PSP-4-Zr-BDPC (BPDC/PSP-1 ∼ 1.5:1)> PSP-2-Zr-BDPC (BPDC/PSP-1 ∼ 1:1)> PSP-1-Zr-BDPC­(BPDC/PSP-1∼ 0.5:1)> PSP-3-Zr-BDPC­(BPDC/PSP-1 ∼ 1:0).

The XRD analysis inferred the higher crystallinity for these heteroleptic materials in comparison to those of **Zr-PSPs** (*vide supra* sections 2.1 and 2.2). The diffraction patterns showed the presence of two intense peaks at low angles (2θ < 10 °) for all materials, characteristic of **Zr-BPDC**-based systems with the UiO-67 topology (see gray pattern in Figure 7a). Shifts of the peaks were noted for the heteroleptic P**SP-1–4-Zr-BPDC**, as well as the appearance of a new reflection at angles slightly higher than the first peak, suggesting notable changes in the structure of the coordination polymers with respect to amorphous **Zr-PSP-1–4** or crystalline **Zr-BPDC** under similar synthetic conditions. In addition, the relative intensities between the first and second peaks are lower than those in Zr-BPDC (the first being more intense than the second) upon the incorporation of **PSP-1**, **PSP-2**, and **PSP-4**. The higher intensity of the peaks in the 5–10° range (see inset in Figure 7a) for the coordination polymers containing amino acids with aromatic side chains (*e.g*., Phe, Tyr, Trp) suggested a positive effect of the π-π attractive interactions on the ordered self-assembly of the coordination polymer.

**Figure 7:**
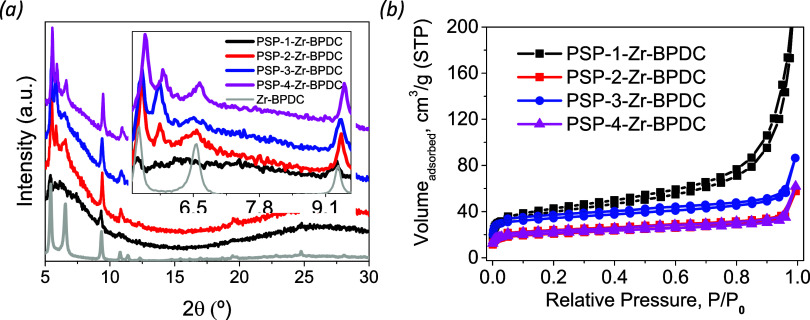
XRD (a) and N_2_-physisorption isotherms (b) of the **PSP-1–4-Zr-BPDC** prepared by the SALE approach.

The N_2_ physisorption isotherms revealed the higher porosity of **PSP-1–4-Zr-BPDC** with respect to **Zr-PSP**-**1–4** structures (Figure 7b), but also with **PSP-1-Zr-BPDC** presenting the highest porosity, probably due to the lower packing efficiency of the valine side chains. The surface areas decreased in the order: **PSP-1-Zr-BDPC** (144 m^2^·g^–1^)> **PSP-2-Zr-BDPC** (78 m^2^·g^–1^)> **PSP-3-Zr-BDPC** (130 m^2^·g^–1^)> **PSP-4-Zr-BDPC** (79 m^2^·g^–1^). In the case of the coordination polymers with aromatic side chains, a higher porosity for the tyrosine containing **PSP-3-Zr-BDPC** was observed, likely as a result of the Bronsted acidic phenol groups that gave a higher number of defects in the 3D arrangement (in line with the lo.) Finally, the SEM images of the different **PSP-1–4-Zr-BPDC** coordination polymers indicated the presence of nanoparticles of regular size and shape, with the biggest nanoparticles being observed for the tryptophan-containing one (Figure 8). **PSP-4-Zr-BPDC** and **PSP-2-Zr-BPDC** nanoparticles seemed more defined and crystalline than those of **PSP-1-Zr-BPDC** and **PSP-3-Zr-BPDC** (more blurry and imperfect), in agreement with the XRD analyses (see insert in Figure 7a).

**Figure 8:**
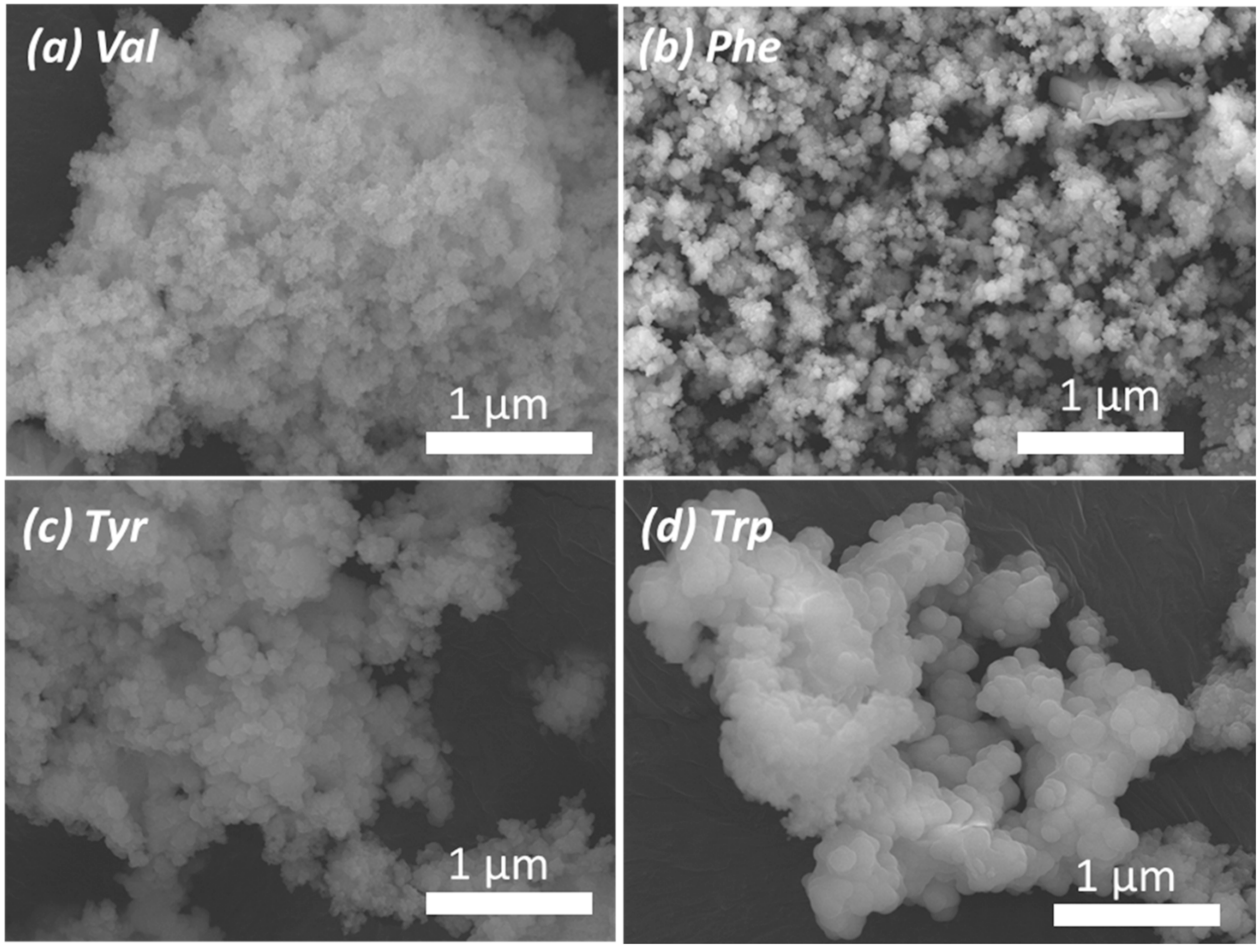
SEM images of the PSP-1-Zr-BPDC (a), PSP-2-Zr-BPDC (b), PSP-3-Zr-BPDC (c), and PSP-4-Zr-BPDC (d) coordination polymers prepared with different amino acids (see 1–4 in Scheme 2) in the presence of formic acid and Zr-BPDC (BPDC = biphenyldicarboxylic acid).

We want to stress that the solvent-assisted linker exchange (SALE) method allows for postsynthetic modification of MOFs by replacing linkers but has limitations. These include heterogeneity in linker distribution, which can affect structural integrity and performance, and incomplete exchange of BPDC by PSP, leading to a mix of modified and unmodified regions. Both issues can impact the MOF’s stability, porosity, and adsorption properties.

### Catalytic Performance of Zr-PSPs in the Activation of Carbonyls

The catalytic performance of the different materials was then investigated in the hydrolysis of p-nitrophenylacetate (**PNPA**) at room temperature and slightly basic pH (pH = 7.5).^38^ We envisaged that the presence of Lewis acid (*i.e*., Zr^4+^ sites) and basic (*i.e*., Zr–O, OH^–^ and COO^–^) groups should activate the ester group and act as nucleophiles in the transformation, respectively. Besides, the electron-poor aromatic diimide cores may also catalyze the hydrolytic reaction by further stabilization of the intermediates/transition states through attractive π-π and anion-π interactions. The reaction course (*i.e*., kinetic profiles) was easily followed by ultraviolet (UV)–vis spectroscopy and the **PNP** yields were determined using a calibration curve (Figures S12–16). Initially, the catalytic activity of **Zr-PSP-1**, prepared with different acids (HFor, HAc, and HCl) as growth modulators (please refer to section 2.1), was evaluated. The use of **Zr-PSP-1-HFor** resulted in a 5-fold increase in rates (24.2 vs 5.7 μM·h^–1^ for **Zr-PSP-1-HFor** and blank, respectively; Figure 9 a, c). The activity seemed to be directly related to the amount of **Zr-PSP** bonds since the materials with higher amounts of free carboxylic acid groups (according to XPS analysis) exhibited poorer catalytic performance.

**Figure 9:**
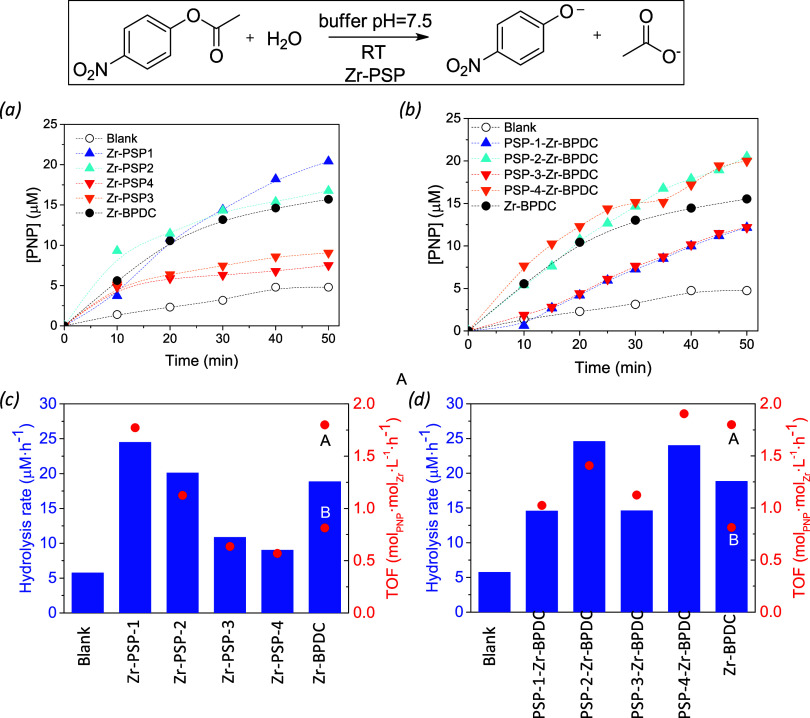
Catalytic performance of Zr-PSP-1–4 (a, c) and PSP-1–4-Zr-BPDC (b, d) samples (prepared from the four amino acids) at the room temperature hydrolysis of p-nitrophenylacetate ester (see Supporting Information for details).

To gain additional insights, we also assayed the effect of the amino acid side chains (and the corresponding changes in porosity/morphology) on the catalytic performance (**Zr-PSP-1–4** systems). The activity followed the order **Zr-PSP-1** > **Zr-PSP-2 > Zr-PSP-3 ≈ Zr-PSP-4**, in agreement with the BET surfaces determined for each material (Figure 10a). Remarkably, despite the much lower porosity of these coordination polymers (<100 m^2^·g^–1^) when compared to well-established MOFs (>1000 m^2^·g^–1^), the activity of **Zr-PSP-1** and **Zr-PSP-2** surpassed that of UiO-67:24.2 and 19.9 vs 18.6 μM·h^–1^, respectively. Benchmark MOFs were also tested as catalysts in the same reaction conditions, and lower amounts of p-nitrophenolate were detected after 50 min with respect to the 20 μM obtained with the Zr coordination polymers: 10.9 μM (ZIF-8) > 9.2 μM (MOF-808) > 3.2 μM (MIL-101) as indicated in Figure S17a. The similar catalytic activity of basic (ZIF-8) or acid (MOF-808) MOFs, both with higher porosity than Zr-PSPs, suggests that additional parameters besides acid–base properties and porosity play a role in the catalytic activity of the pseudopeptidic Zr coordination polymers. One of those might be H-bonding activation by the naphthalene diimide backbone, which might enhance the nucleophilicity of the water reagent at the Zr-PSP catalytic pockets.

**Figure 10:**
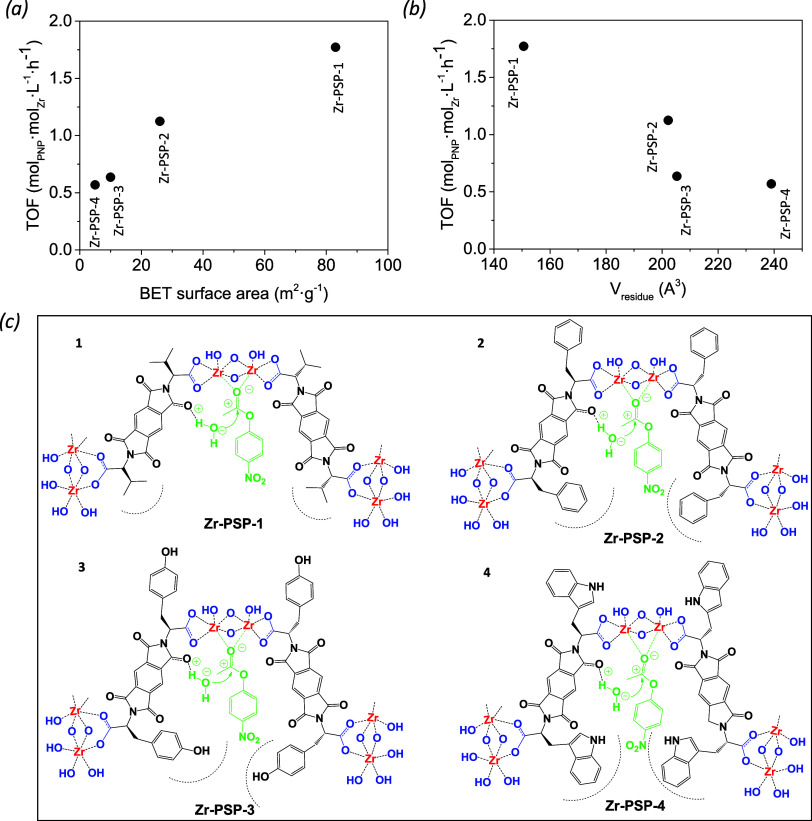
Catalytic performance (expressed as the TOF of the Zr sites present) of Zr-PSP samples prepared from the four amino acids at room temperature, the hydrolysis of esters with respect to their surface area (a), amino acid residue volume (b), and the confined transition states proposed (c).

All Zr-PSP samples exhibited a pH < 5.5 when suspended in water, thus releasing protons from polarized water molecules at the strong Zr Lewis acid sites. When aqueous (nonbuffered) solutions of **PNP** (deprotonated in deionized water at pH 7) are put in contact with the **Zr-PSP-1**, the protonation of the phenolate groups takes place, further proving the acidity of the Zr sites present at the coordination polymer (see Figure S18c). However, no direct correlation between acid strength (pH decrease) and catalytic activity is observed, indicating that other factors control the rate in the porous materials (Figure S18a). As commented for the Zr-PSP-1 sample prepared in the presence of different acids, one of these is the amount of PSP coordinated to Zr. For the four samples of Zr-PSP1–4, an increase in catalytic activity is observed when the PSP/Zr ratio is increased (see Figure S18b). Furthermore, the presence of phthalamide can alter the environment of Zr, forming hydrogen bonds with the substrate, similar to an enzymatic system that can vary the activity of the system (see Figure 10). This is not observed in the other MOFs tested, *i.e*., ZIF-8, MOF-808 and MIL-101, being a plausible cause of their lower activity despite their higher porosity. Therefore, the difference with Zr-based MOFs such as UiO-67 or MOF-808 is not that the Zr is more acidic, but rather its high catalytic activity is due to the presence of hydrogen bonds and additional interactions introduced by the amino acids, as compared to the ligands in conventional MOFs.

Note that the reaction rate is expressed as the amount of PNPA converted into PNP per hour (μM_PNP_·h^–1^), and the TOF of each catalyst can be estimated considering the amount of to the Zr sites present in the solid (obtained from the ZrO_2_ amount of the TGA); the activity increases in the following order: Zr-PSP-4 (0.57 mol_PNP_·mol_Zr_·h^–1^) < Zr-PSP-3 (0.64 mol_PNP_·mol_Zr_·h^–1^) < Zr-PSP-2 (1.12 mol_PNP_·mol_Zr_·h^–1^) < Zr-PSP-1 (1.77 mol_PNP_·mol_Zr_·h^–1^). This suggests the negative effect on the catalytic activity of the steric hindrance of bulk lateral chains of the amino acids (especially that of phenol and indol-like side chains in Zr-PSP-2 and Zr-PSP-3, respectively) and diffusion control of the reaction at the pores of the solid (being controlled by the polarity and π-π electron-rich interaction of the amino acid side chain with the diimide cores from the PSP). It is worth mentioning the high catalytic activity of the Zr-PSP concerning traditional Zr-MOFs such as Zr-BPDC (UiO-67, using two different samples with different amounts of BDC incorporated, A and B in Figures 9b,d andS11), which have much higher surface areas (>1000 m^2^·g^–1^). In Figure 10, we have represented the parallel trend between catalytic activity and both specific surface BET area (a) and volume of the amino acid residues (b) present in the four Zr-PSP samples, indicating the key role of the amino acid side chain volume in the porosity and thus catalytic performance of the material. Figure 10c represents the confinement of the reagents inside the Zr-PSP pores and the steric hindrance of the amino acid tyrosine and tryptophan residues.

In the case of the **PSP-Zr-BPDC** (please refer to section 2.3), a similar TOF to that obtained with pristine **Zr-BPDC** (0.8–1.7 mol_PNP_·mol_Zr_·h^–1^, using two different samples, A and B in Figures 9d andS11) was achieved with all of the **PSP-Zr-BPDC** samples (1.0–1.9 mol_PNP_·mol_Zr_·h^–1^). Probably, the higher incorporation of PSP-1 (with respect to BPDC) within the porous Zr-BPDC framework blocks part of the porosity/activity of such matrix, resulting in lower values of TOF concerning bulk Zr-PSP-1 (1.0 vs 1.78 mol_PNP_·mol_Zr_·h^–1^, respectively). In contrast to the bulk Zr-PSPs, no direct trend between the porosity and the activity of the PSP-Zr-BPDC materials was found, suggesting that other factors influence their catalytic performance (see Figure 11a). PSP1-Zr-BPDC and PSP-3-Zr-BPDC exhibit smaller XRD peaks, especially for the first peak that appears to be shifted concerning those of Zr-BPDC, probably negatively affecting its ordered structure and thus accounting for its lower catalytic activity (*ca.* 0.9–1.0 mol_PNP_·mol_Zr_·h^–1^). On the other hand, Figure 11 b shows that the poor incorporation of the valine-containing PSP-1 and tyrosin-containing PSP-3 with respect to PSP-2 and PSP4 (PSP/BPDC ratio obtained from the NMR of the digested samples) is key in the catalytic activity (*ca.* 1.1 vs 1.4/1.9 mol_PNP_·mol_Zr_·h^–1^, respectively). In this sense, a similar trend is observed for the tryptophan-containing PSP-4-Zr-BPDC, having unexpectedly high catalytic activity (1.9 mol_PNP_·mol_Zr_·h^–1^) compared with bulk Zr-PSP-4 (0.6 mol_PNP_·mol_Zr_·h^–1^). This may also be attributed to the higher incorporation (PSP-4/BPDC ratio of 1.5) and better dispersion of the tyrosine side chains within a more porous framework with respect to Zr-PSP-4 (*ca.* 80 vs 5 m^2^·g^–1^) on the catalytic activity of this multifunctional material. A somewhat similar increase in the catalytic activity of bulk and amorphous Zr-PSP samples with the amount of PSP incorporated (for the same amount of Zr, according to TGA) is also found (see Figure S19b).

**Figure 11:**
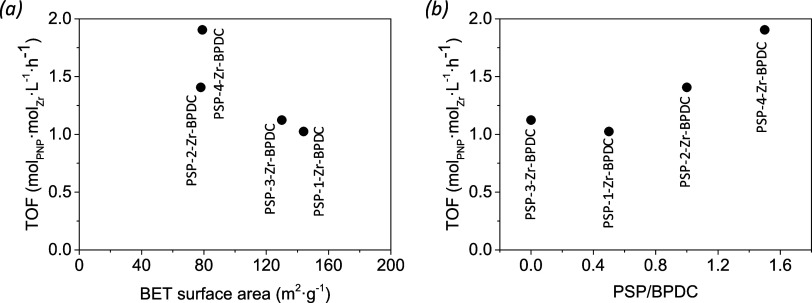
Catalytic performance (expressed as the TOF of the Zr sites present) of PSP-Zr-BPDC samples prepared from the four amino acids at room temperature, the hydrolysis of esters with respect to their surface area (a), and the amount of PSP incorporated (in moles) with respect to BPDC (b).

Although the coordination polymers lack long-range crystallinity, which prevents monitoring structural integrity *via* PXRD, no leaching of Zr or PSP ligands was detected in the reaction solution after catalysis, as confirmed by ICP and UV–vis analyses (see Figures S13–S14). This supports the stability of the Zr–O coordination framework, which is also consistent with the high thermal robustness observed in the TGA profiles. Regarding recyclability, we performed multiple catalytic cycles under identical conditions (Figure S17b). While the material retained some catalytic activity, a gradual decrease in performance was observed over successive cycles. This deactivation is likely due to pore plugging or surface fouling, which is plausible considering the inherently low porosity of the Zr-PSP materials.

## Conclusions

A pseudopeptidic linker (**PSP**) based on functionalized pyromellitic anhydride and amino acids (1–4) was prepared under microwave conditions. The synthesis and characterization of the PSP coordination polymers containing different amino acid residues from valine (1), phenylalanine (2), tyrosine (3), and tryptophan (4) are reported using both zirconium chloride salt and Zr-BPDC as metal sources. The best conditions for the formation of bulk **Zr-PSP-1** from ZrCl_4_ and different acids as nanoparticle growth modulators have been studied by FTIR, XRD, XPS, TGA, and SEM analysis. The optimal conditions (use of formic acid as modulator) were employed to obtain the bulk **Zr-PSP-1–4** and study the composition, geometry, and porosity of the resulting coordination polymers. On the other hand, the PSPs were also incorporated in a defective preformed Zr-BPDC under the same synthesis conditions of the pseudopeptidic MOFs in one pot, indicating significant changes in the composition and crystalline structure of pristine Zr-BPDC. In both cases, regular porous nanoparticles with a hybrid organic–inorganic composition were obtained, with (PSP-Zr-BPDC) or without (Zr-PSP) a crystalline structure. The catalytic activity of new materials is evaluated in the hydrolysis of an ester (p-nitrophenylbenzoate methyl ester), where the PSP incorporation (for both PSP-Zr-BPDC and Zr-PSP) and porosity (especially for Zr-PSP) are key properties in the activity of the porous coordination polymers. The new materials show a competitive behavior with respect to reported Zr-BPDC, exhibiting the possibility of tuning the structure (and thus performance) by the change in the amino acid residue (volume and polarity), reaching hydrolysis rates of up to 30 μM·h^–1^ and TON values of up to 2 mol_PNP_·mol_Zr_·h^–1^.

## Supplementary Material



## Notes

Additional experimental details; materials, and methods; including the synthesis; characterization, and catalysis (PDF)
