# Supplementary material for: Pseudopeptidic Coordination Polymers Based on Zirconium-Carboxylate Supramolecular Assemblies
Source: ACS Appl Mater Interfaces. 2025 May 5;17(19):28555–67. doi: 10.1021/acsami.5c02827 (PMC12129261; doi:10.1021/acsami.5c02827)
Supplement: Supplementary file 1 [file am5c02827_si_001.pdf]

SUPPORTING INFORMATION

Pseudopeptidic Coordination Polymers based on Zirconium-Carboxylate Supramolecular Assemblies

Miguel Maireles-Porcar±,[a] Ferran Esteve±,[a,b] Nuria Martín,[a] Julian Sanchez Velandia,[a] Belén Altava,[a] Francisco G. Cirujano,[a*] Eduardo García-Verdugo[a*]

[a] Departamento de Química Inorgánica y Orgánica, Universitat Jaume I. Av. Vicent Sos Baynat, s/n, 12006. Castelló de la Plana, Castelló. España.
[b] Laboratoire de Chimie Supramoléculaire, Institut de Science et d'Ingénierie Supramoléculaires (ISIS), Université de Strasbourg, 8 allée Gaspard Monge, 6700. Strasbourg, France.
±These authors contributed equally
cepeda@uji.es; cirujano@uji.es

INDEX

S1. SYNTHESES OF LINKERS……………………………………………………..page 2

S2. SYNTHESIS AND CHARACTERIZATION OF Zr-PSP-1……..……………..page 6

S3. SYNTHESIS AND CHARACTERIZATION OF Zr-PSPs WITH DIFFERENT AMINO ACIDS……………………………………………………………………..page 11

S4. SYNTHESIS AND CHARACTERIZATION OF PSP-1/UiO-66 BY PSP INCORPORATION INTO A PREFORMED Zr-BPDC MOF……………………page 14

S5. CATALYTIC PERFORMANCE ………………………………………………..page 16

S1. SYNTHESES OF LINKERS

PSP-1: 1 g (8,5 mmol) of L-valine and 1 g (4,5 mmol) of pyromellitic dianhydride were weighted in a microwave tube, dissolved in 5 mL of acetic acid and placed in the microwave oven at 200 W, 160 ºC, 200 PSI for 0.5 h. The resulting grey solution was cooled to room temperature and the pseudopeptide N,N′-bis(L-valine)pyromellitic diimide linker water was precipitated after the addition of water. The solid was filtered and washed by redissolving in THF and subsequently evaporated under vacuum, resulting in a crystalline white solid (weight 1.44g). N,N′-bis(L-valine)pyromellitic diimide, $C_{20}N_2O_8H_{18}$, MW = 416.4 g·mol^{-1} (PSP-1): FTIR 3211, 2966, 1703, 1375, 1358 y 1278 cm^{-1}; ^{1}H NMR (400 MHz, DMSO) δ 13.10 (s, 2H), 8.33 (s, 2H), 4.54 (d, J = 7.8 Hz, 2H), 2.70-2.54 (m, 2H), 1.08 (d, J = 6.7 Hz, 6H), 0.85 (d, J = 6.8 Hz, 6H).

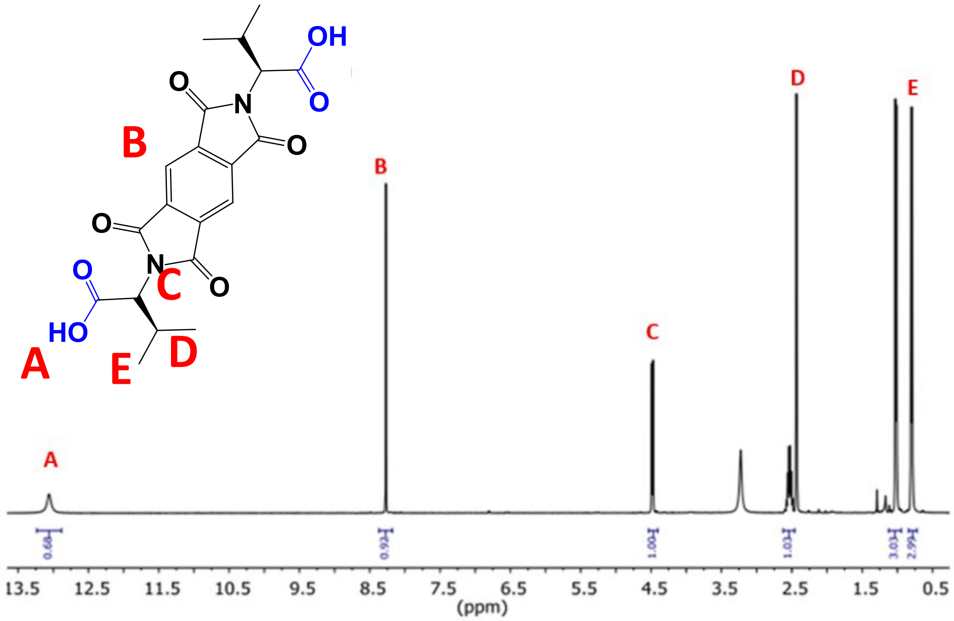

Figure S1. H-NMR spectra of the pseudopeptidic linker **PSP-1**, indicating the H characteristics in the molecular structure.

PSP-2: 1 g (6.1 mmol) of L-phenylalanine (Phe) and 0.65 g (3 mmol) of pyromellitic dianhydride were weighed in a microwave tube, dissolved in 5 mL of acetic acid and placed in the microwave oven at 200 W, 160 ºC, 200 PSI for 0.5 h. The resulting grey solution was cooled to room temperature and the pseudopeptide N,N′-bis(L-phenylalanine)pyromellitic diimide linker water was precipitated after adding water. The solid was filtered and washed by redissolving in THF and subsequently evaporated under vacuum, resulting in a crystalline white solid (weight 0.051g). N,N′-bis(L-phenylalanine)pyromellitic diimide $C_{26}N_2O_8H_{16}$, MW = 484.4 g·mol^{-1} (PSP-2): FTIR 2940, 2365, 1715, 1381 y 1365 cm^{-1}; ^{1}H NMR (400 MHz, DMSO) δ 8.21 (s, 2H), 7.21 – 7.10 (m, 10H), 5.15 (dd, J = 11.3, 4.8 Hz, 2H).

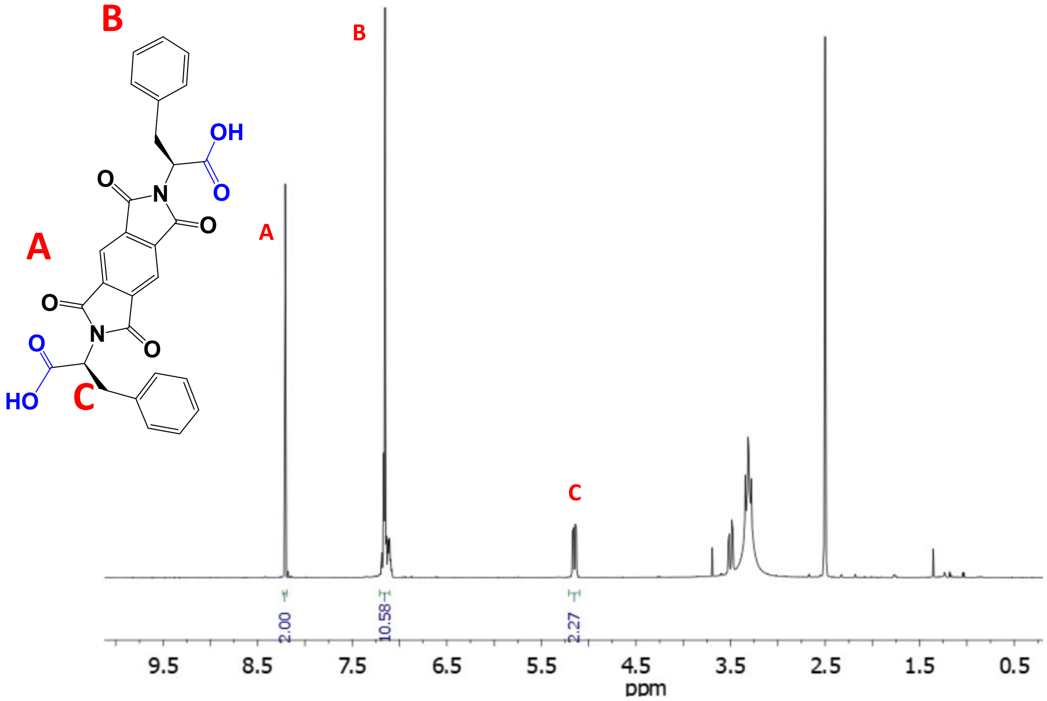

Figure S2. H-NMR spectra of the pseudopeptidic linker **PSP-2**, indicating the H characteristics in the molecular structure.

PSP-3: 1.01 g (5.5 mmol) of L-tyrosine (Tyr) and 0.60 g (2.75 mmol) of pyromellitic dianhydride were weighed in a microwave tube, dissolved in 5 mL of acetic acid and placed in the microwave oven at 200 W, 160 ºC, 200 PSI for 0.5 h. The resulting grey solution was cooled to room temperature and the pseudopeptide N,N′-bis(L-tyrosine)pyromellitic diimide linker was precipitated after the addition of water. The solid was filtered and washed by redissolving in THF and subsequently evaporated under vacuum, resulting in a crystalline white solid (weight 0.539g). N,N′-bis(L-tyrosine)pyromellitic diimide $C_{26}N_2O_{10}H_{16}$, MW = 516.4 g·mol^{-1} (PSP-3): FTIR 3350, 1706, 1514 1384, 1365 cm^{-1}; ^{1}H NMR (400 MHz, DMSO) δ 12.96 (s, 2H), 9.14 (s, 2H), 8.23 (d, J = 1.2 Hz, 2H), 6.93 (d, J = 8.4 Hz, 4H), 6.54 (d, J = 8.4 Hz, 4H), 5.06 (dd, J = 11.3, 4.8 Hz, 2H). ^{13}C NMR (75 MHz, DMSO) δ 169.6 (s), 165.2 (s), 155.8 (s), 136.2 (s), 129.6 (s), 127.0 (s), 118.5 (s), 115.2 (s), 53.9 (s), 40.3 (s), 40.0 (s), 39.8 (t, J = 19.4 Hz), 39.4 (d, J = 14.2 Hz), 39.1 (d, J = 14.2 Hz), 38.9 (s), 38.7 (s), 33.0 (s), 21.0 (s).

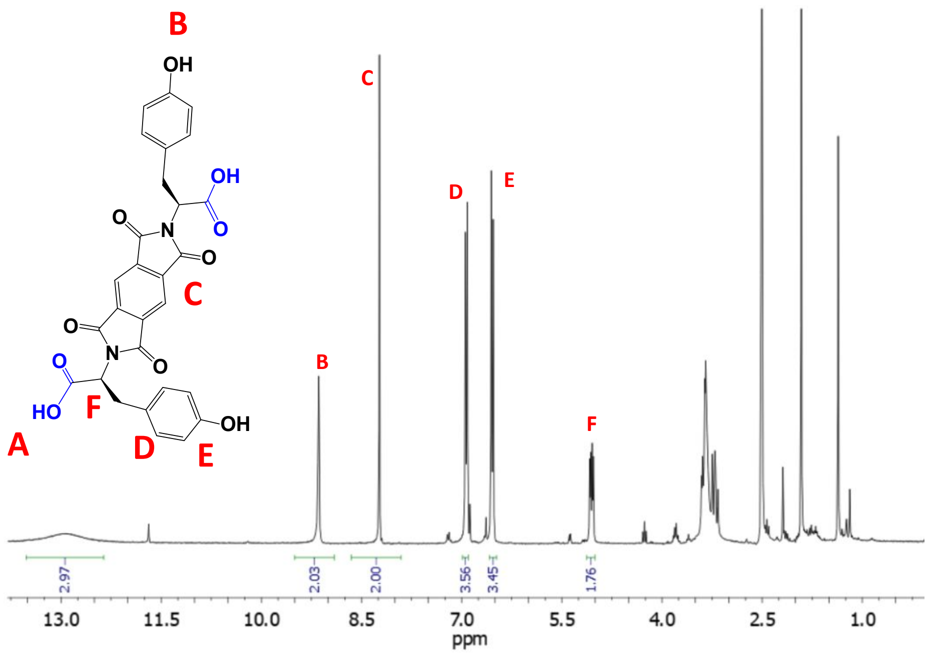

Figure S3. H-NMR spectra of the pseudopeptidic linker **PSP-3**, indicating the H characteristics in the molecular structure.

PSP-4: 0.99 g (4.9 mmol) of L-tryptophan (Trp) and 0.603 g (2.45 mmol) of pyromellitic dianhydride were weighed in a microwave tube, dissolved in 5 mL of acetic acid and placed in the microwave oven at 200 W, 160 ºC, 200 PSI for 0.5 h. The resulting grey solution was cooled to room temperature and the pseudopeptide N,N′-bis(L-tryptophan)pyromellitic diimide linker was precipitated after the addition of water. The solid was filtered and washed by redissolving in THF and subsequently evaporated under vacuum, resulting in a crystalline white solid (weight 0.462g). N,N′-bis(L-tryptophan)pyromellitic diimide $C_{32}N_4O_8H_{22}$, MW = 590.4 g·mol^{-1} (PSP-4): FTIR 3403, 1712, 1384, 1364, 1231, 1107 cm^{-1}; ^{1}H NMR (400 MHz, DMSO) δ 13.35 (s, 2H), 10.75 (d, *J* = 16.1 Hz, 2H), 8.21 – 8.11 (m, 1H), 7.53 – 7.41 (m, 2H), 7.23 (t, *J* = 8.4 Hz, 4H), 7.09 – 6.95 (m, 4H), 6.95 – 6.81 (m, 4H), 5.30 – 5.22 (m, 1H), 5.14 (dd, *J* = 10.2, 5.5 Hz, 2H), 3.75 – 3.46 (m, 6H), 1.91 (s, 1H), 1.84 – 1.66 (m, 2H), 1.36 (s, 1H). ^{13}C NMR (75 MHz, DMSO-d) δ 169.8 (s), 165.2 (s), 136.2 (s), 136.0 (s), 126.7 (s), 123.5 (s), 120.9 (s), 118.3 (d, *J* = 11.2 Hz), 117.8 (s), 111.4 (s), 109.4 (s), 53.2 (s), 40.3 (s), 39.9 (d, *J* = 21.0 Hz), 39.6 (s), 39.4 (d, *J* = 18.1 Hz), 39.2 (s), 39.1 (d, *J* = 21.0 Hz), 38.6 (s), 24.0 (s).

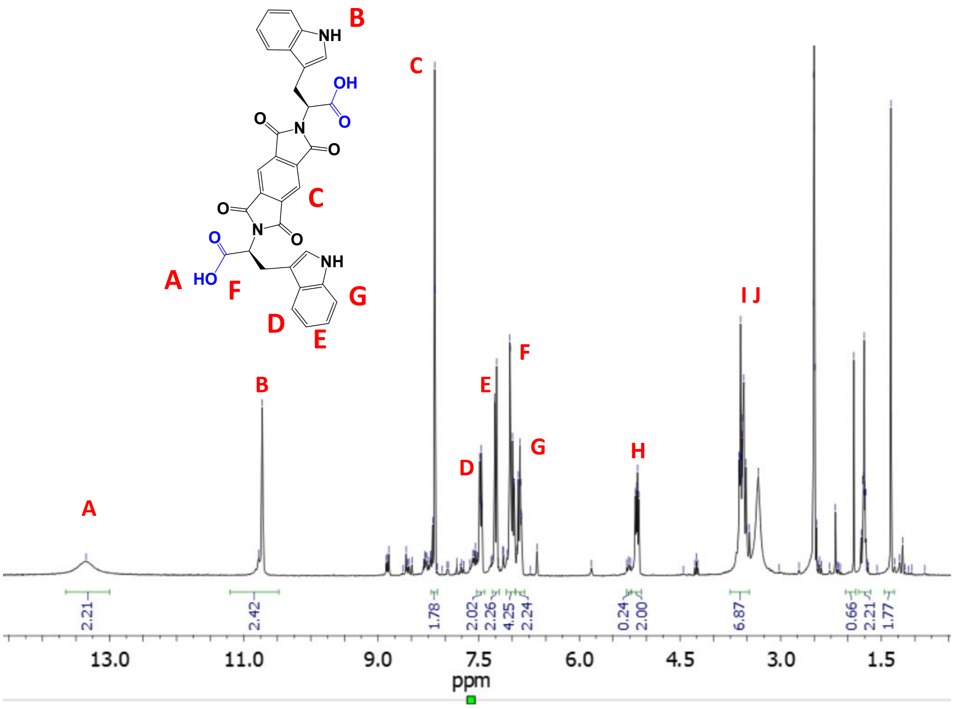

Figure S4. H-NMR spectra of the pseudopeptidic linker **PSP-4**, indicating the H characteristics in the molecular structure.

S2. SYNTHESIS AND CHARACTERIZATION OF Zr-COORDINATION POLYMERS

Synthesis of Zr-PSP: In a 50 mL Schott glass bottle, 53 mg (0.23 mmol) of $ZrCl_4$ and 95 mg (0.23 mmol) of PSP-1 were dissolved by using 9 mL of DMF and 1 mL of acid (see HA in Scheme 1b). Based on the positive effect of acids as modulators of the crystal growth in Zr-MOF systems, different acids were employed as modulators for the growth of the Zr-PSP-1, both organic (acetic and formic acids) or mineral (hydrochloric acid), as indicated in Scheme 1 (see HX, where X = acetate, formate, and chloride). The solution was placed in an oven at 120 ºC for 1 day and the withe Zr-PSP-1A, B or C (depending on the acid employed, A with acetic acid, B with hydrochloric acid and C with formic acid) powder was formed. The bottle was cooled to room temperature, the powder was isolated by centrifugation, washed with methanol and dried overnight (weight = 92 mg). FTIR 2966, 1721, 1650, 1600, 1378, 1350 cm^{-1}; The synthesis of Zr-PSP-1-4-HFor coordination polymers was done similarly. Briefly, in a 50 mL Schott glass bottle, 53 mg (0.23 mmol) of de $ZrCl_4$ and either 117 mg (0.23 mmol) of PSP-2, 127 mg (0.23 mmol) of PSP-3 or 150 mg (0.23 mmol) of PSP-4 were dissolved by using 9 ml of DMF and 1 ml of formic acid. The solution was placed in an oven at 120 ºC for 1 day and the white (Zr-PSP-2-HFor) or yellow (Zr-PSP-3-HFor) or brown (Zr-PSP-4-HFor) powder was formed. The bottle was cooled to room temperature, the powder was isolated by centrifugation, washed with methanol and dried overnight.
Zr-PSP-2-HFor (weight 62mg) FTIR 3253, 1715, 1579, 1423, 1370 cm^{-1}
Zr-PSP-3-HFor (weight 68mg) FTIR 1712, 1580, 1412, 1369 cm^{-1}
Zr-PSP-4-HFor (weight 71mg) FTIR 3250, 1712, 1579, 1412, 1367 cm^{-1}

Synthesis of Zr-PSP-BPDC:
PSP-BPDC mix-linker synthesis approach: 53 mg (0.23 mmol) of $ZrCl_4$, 77 mg (0.184 mmol) of PSP-1 and 13 mg (0.046 mmol) of BPDC were dissolved by using 9 ml of DMF and 1 ml of acetic acid. The solution was placed in an oven at 120 ºC for 1 day and the powder was formed. The bottle was cooled to room temperature, the powder was isolated by centrifugation, washed with methanol and dried overnight.
The solvent-assisted linker exchange (SALE) approach was performed in two different ways: (a) 200 mg of PSP-1 dissolved in 3 mL of DMF (in an ultrasound bath for 5 min at RT) was added to UiO-67 (50mg) placed in a glass vessel. The suspension was sonicated for 5 min at room temperature. and placed in the oven at 120ºC for 10 days. After that, the solid was recovered by centrifugation and washed three times with DMF and dried under vacuum at 60ºC. (b): 105 mg of $ZrCl_4$ and 23 mg of BPDC (20% mol with respect to Zr) were dissolved in 18 mL of DMF and 2 mL of HFor (ultrasound 5 min RT). The suspension was heated in an oven at 120ºC for 24h. After that, the reaction vessel was cooled to room temperature, and 1 eq. of PSP-1 (180 mg) was added and left for another 24h. This operation was repeated twice. The solid was isolated by filtration and washed three times with DMF to remove the PSP that is not incorporated into the solution.

Synthesis of benchmark MOFs

MIL-101-Fe-NH$_2$: In a 50 mg flask, 679.3 mg of $FeCl_3$ and 226 mg of 2-amino terephthalic acid are dissolved in 18 mL of DMF. The flask is heated at 120°C for 24 hours, and the mixture is centrifuged to remove the solvent. The solid is washed twice with fresh DMF and twice with EtOH. The product is dried in a vacuum oven for 24 hours.

MOF-808: In a 250 mL flask, 265 mg of $ZrCl_4$ and 82 mg of trimesic acid are dissolved in 25 mL of DMF and 25 mL of HCOOH. The solution is heated at 120°C for 24 hours, and then the mixture is centrifuged to separate the solid phase from the liquid phase. The solid is washed twice with fresh DMF and twice with MeOH. The product is dried in a vacuum oven for 24 hours.

ZIF-8: In a 250 mL round-bottom flask, 1.75 g of $Zn(NO_3)_2 \cdot 6H_2O$ is dissolved in 30 mL of miliQ H_2O. In another 250 mL beaker, 3.2 mg of 2-methylimidazole is dissolved in 30 mL of H_2O and 5 mL of triethylamine. The solution containing the ligand is added dropwise to the one containing the metal. The reaction takes place at room temperature for 1 hour. After the time has passed, the mixture is centrifuged to separate the solid phase from the liquid phase. The solid is washed with MeOH and dried in a vacuum oven for 24 hours.

S3. CHARACTERIZATION OF THE COORDINATION POLYMERS

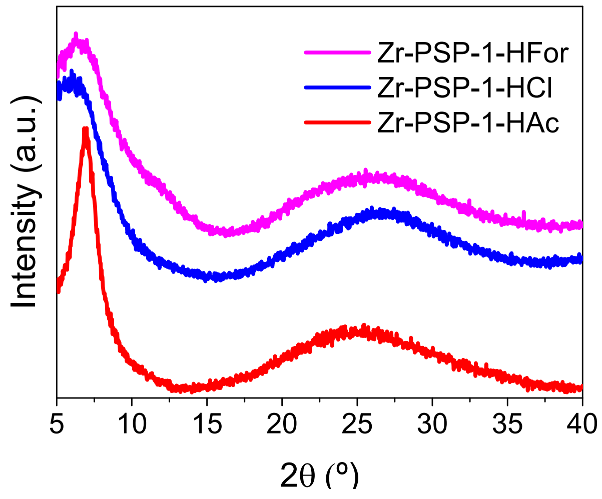

Figure S5. XRD analysis of samples **Zr-PSP-1-HAc** (red line), **Zr-PSP-1-HCl** (blue line), **Zr-PSP-1-HFor** (purple line).

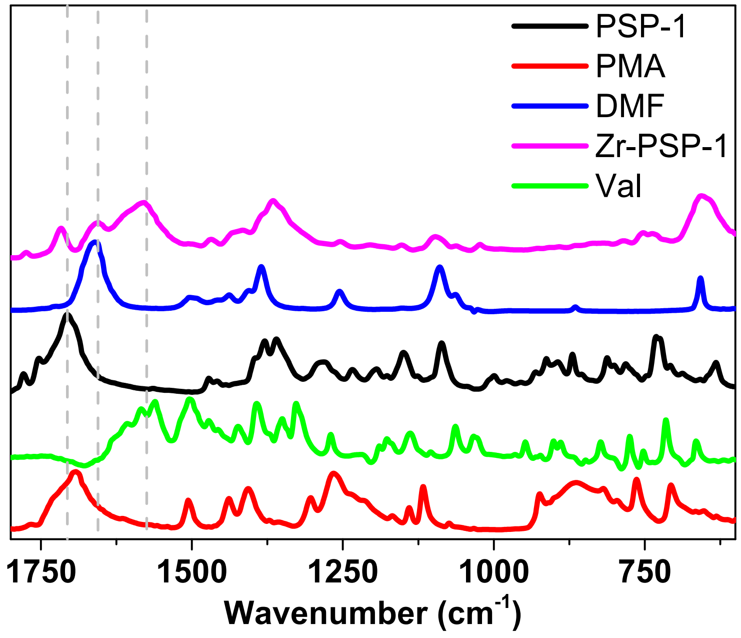

Figure S6. FTIR analysis of samples **Zr-PSP-1-HFor** (purple line), DMF (blue line), **PSP-1** (black line), Valine (red line) and pyromelitic dianhydride (red).

Table S1. FTIR analysis of the **Zr-PSP-1** coordination polymers prepared in the presence of acetic (HAc), hydrochloric (HCl) and formic (HFor) acids, as well as in the presence of other amino acids (**Zr-PSP-2-HFor**, **Zr-PSP-3-HFor**, **Zr-PSP-4-HFor**).

Sample	Area(COOH)[a]	Area(COOZr)[b]	%(COOH)[c]	%(COO-Zr)[d]
**Zr-PSP-1-HAc**	381	1080	26	74
**Zr-PSP-1-HCl**	672	1402	32	68
**Zr-PSP-1-HFor**	300	1077	21	78
**Zr-PSP-2-HFor**	169	806	17	82
**Zr-PSP-3-HFor**	132	705	16	84
**Zr-PSP-4-HFor**	183	841	18	82

[a] Integral below the curve limited between 1760 and 1685 cm^{-1}; [b] Integral below the curve limited between 1685 and 1490 cm^{-1}; [c] [Area(COOH)/[Area(COOH) + Area(COO-Zr)]]*100; [d] [Area(COOZr)/[Area(COOH) + Area(COOZr)]]*100.

Table S2. TGA analysis (from 25 ºC to 800 ºC (10 ºC/min) under air) of the **Zr-PSP-1** coordination polymers prepared in the presence of acetic (HAc), hydrochloric (HCl) and formic (HFor) acids as well as **Zr-PSP-2-4-HFor**.

Sample	% Guests[a]	% PSP[b]	%ZrO_2[c]	mol_{PSP}/mol_{Zr}
**PSP**	3	97	-	-
**Zr-PSP-1-HAc**	13	12	75	0.05
**Zr-PSP-1-HCl**	61	26	13	0.59
**Zr-PSP-1-HFor**	30	36	34	0.31
**Zr-PSP-2-HFor**	21	35	44	0.20
**Zr-PSP-3-HFor**	24	34	42	0.19
**Zr-PSP-4-HFor**	24	37	39	0.20

[a]weight loss below 150 ºC; [b]weight loss (%) between 300 and 600 ºC; [c]weight loss (%) above 600ºC; [d]both PSP and biphenyldicarboxylic acid are included in this mass loss.

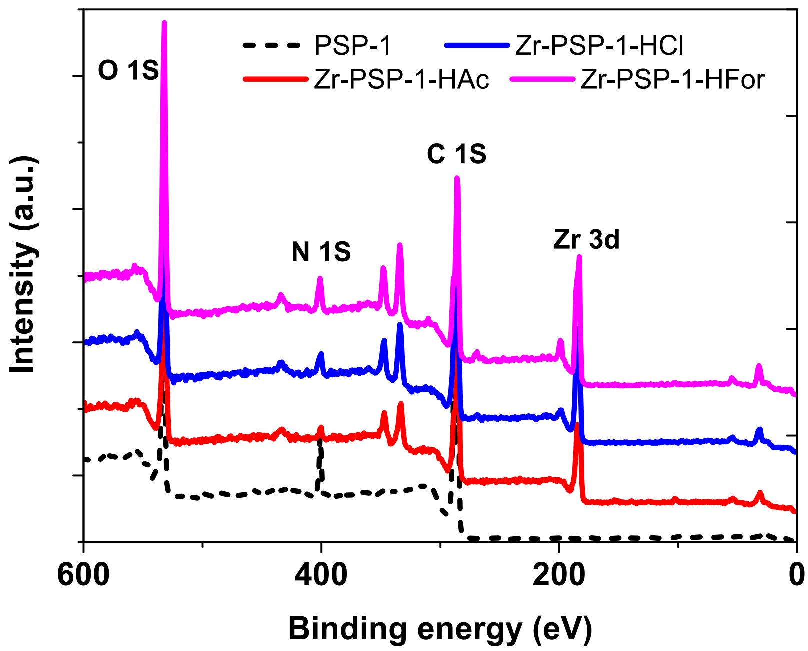

Figure S7. XPS analysis of samples PSP (black dots), **Zr-PSP-1-HAc** (red line), **Zr-PSP-1-HCl** (blue line), **Zr-PSP-1-HFor** (purple line).

Table S3. XPS analysis of the **Zr-PSP-1** coordination polymers prepared in the presence of acetic (HAc), hydrochloric (HCl) and formic (HFor) acids.

Sample	Atomic concentration (%)				Surface Composition
	C	N	O	Zr	
**PSP-1**	69.5	7.3	23.3	-	$C_{7.6}N_1O_{3.2}$
**Zr-PSP-1-HAc**	61.3	2.6	28.2	7.8	$Zr_1C_{7.9}N_{0.3}O_{3.6}$
**Zr-PSP-1-HCl**	57.4	4.9	28.4	9.3	$Zr_1C_{6.2}N_{0.5}O_{3.0}$
**Zr-PSP-1-HFor**	58.1	5.3	27.6	8.9	$Zr_1C_{6.5}N_{0.6}O_{3.1}$

Table S4. XPS (O1s) analysis of the **Zr-PSP-1** coordination polymers prepared in the presence of acetic (HAc), hydrochloric (HCl) and formic (HFor) acids. Binding energies and percentage of deconvoluted bands with respect to the total oxygen (in parenthesis).

Sample	O-Zr (%)	C=O (%)	-O-C=O (%)	-OH (%)
**PSP**	- (0)	531.9 (64.2)	533.2 (26.1)	534.3 (9.7)
**Zr-PSP-1-HAc**	530.2 (16.2)	531.8 (51.2)	533.2 (22.6)	535.0 (9.9)
**Zr-PSP-1-HCl**	530.1 (18.2)	531.7 (56.1)	533.0 (17.5)	534.4 (8.1)
**Zr-PSP-1-HFor**	530.4 (21.3)	531.9 (63.9)	533.5 (14.8)	- (0)

S3. SYNTHESIS AND CHARACTERIZATION OF Zr-PSPs WITH DIFFERENT AMINO ACIDS

Table S5. Surface area (from N_2 physisorption) of the coordination polymers

Sample	Amino acid	BET (m^2/g)
**Zr-PSP-1**	Valine	83
**Zr-PSP-2**	Phenylalanine	26
**Zr-PSP-3**	Tyrosine	10
**Zr-PSP-4**	Tryptophan	5
**PSP-1-Zr-BPDC**	Valine	144
**PSP-2-Zr-BPDC**	Phenylalanine	78
**PSP-3-Zr-BPDC**	Tyrosine	130
**PSP-4-Zr-BPDC**	Tryptophan	79

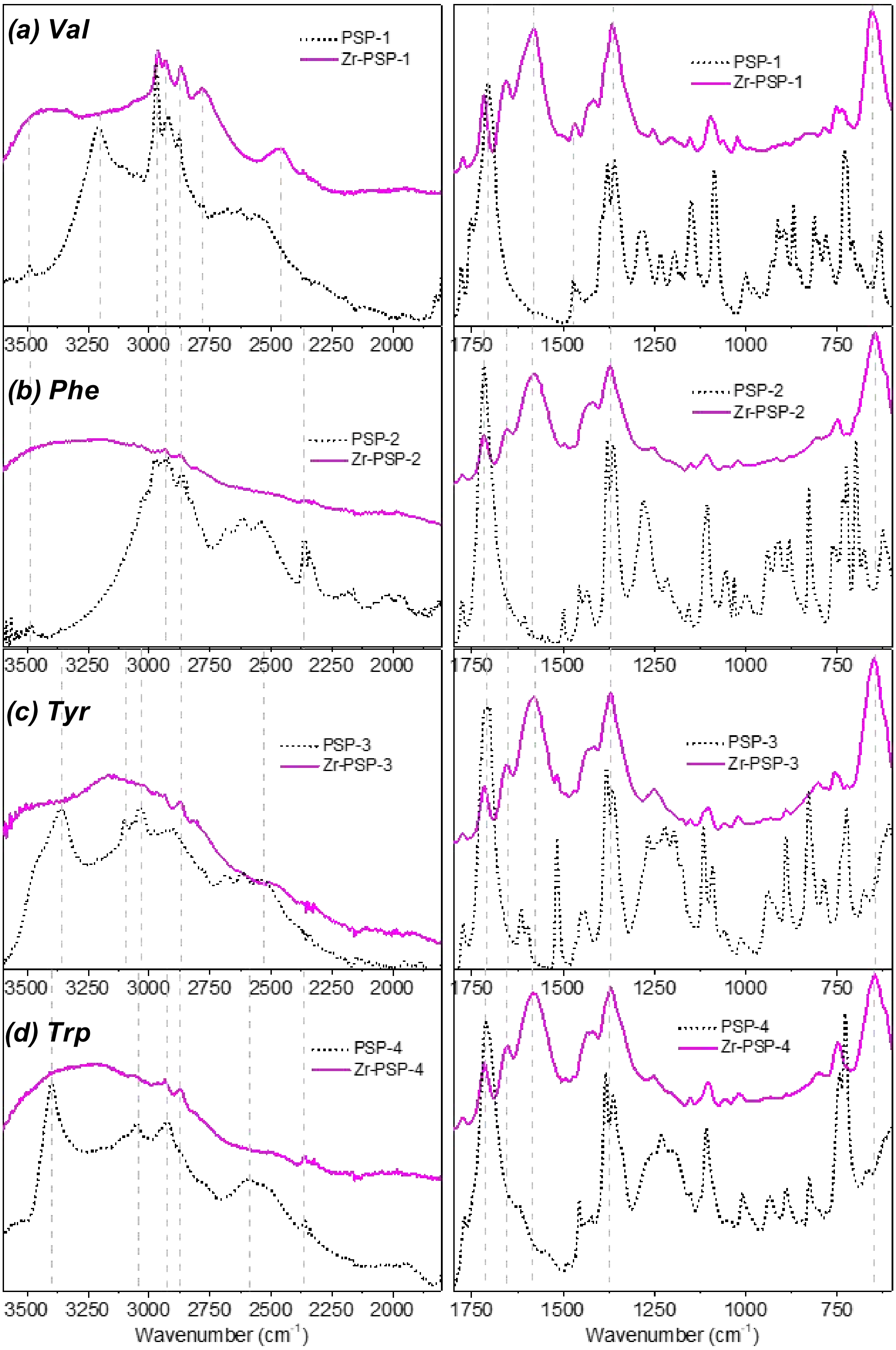

Figure S8. FTIR spectra of the pseudopeptidic linkers (black dotted line) **PSP-1** (a), **PSP-2** (b), **PSP-3** (c), **PSP-4** (d) prepared with different amino acids (see 1-4 in Scheme 2) and **Zr-PSP-1** (a), **Zr-PSP-2** (b), **Zr-PSP-3** (c), **Zr-PSP-4** (d) coordination polymers prepared in the presence of formic acid (magenta line).

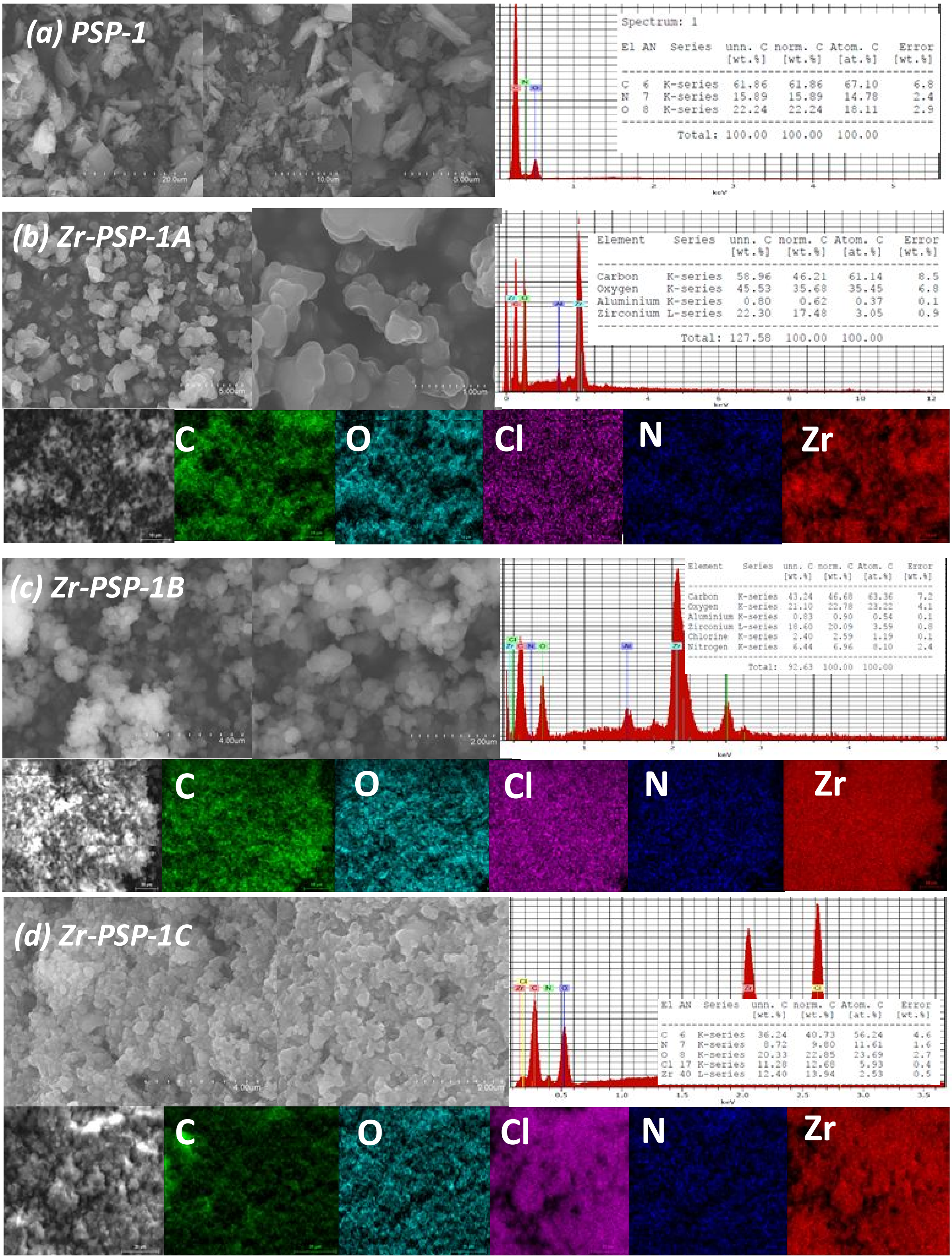

Figure S9. SEM (top-left)-EDX (top-right and bottom) analysis of the **PSP-1** linker (a) and **Zr-PSP-1** coordination polymers prepared in the presence of acetic (b), hydrochloric (c) and formic acid (d).

S4. SYNTHESIS AND CHARACTERIZATION OF PSP-1/UiO-66 BY PSP INCORPORATION INTO A PREFORMED UiO-67 MOF.

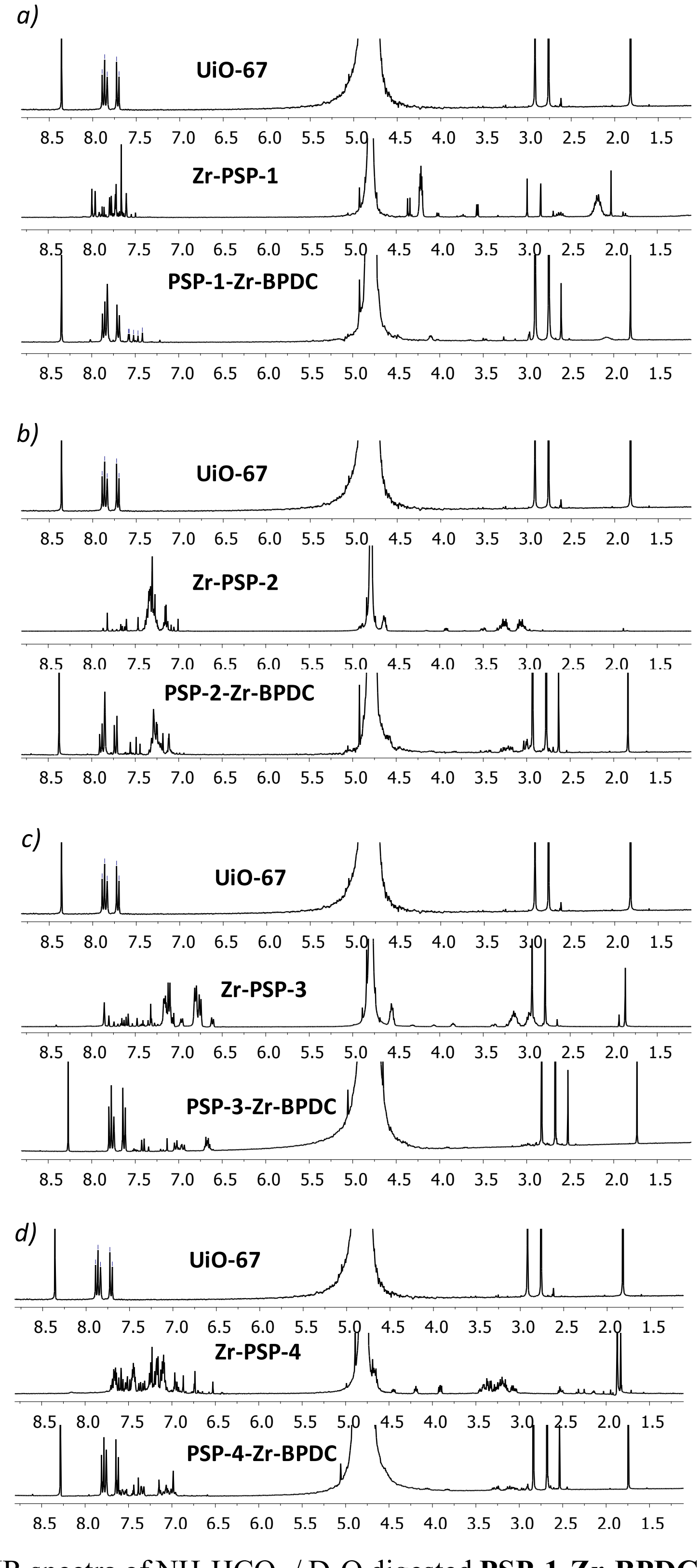

Figure S10. H-NMR spectra of NH_4HCO_3 / D_2O digested **PSP-1-Zr-BPDC** and **Zr-PSP-1** (a), **PSP-2-Zr-BPDC** and **Zr-PSP-2**, (b) **PSP-3-Zr-BPDC** and **Zr-PSP-3**, (c), **PSP-4-Zr-BPDC** and **Zr-PSP-4** (d).

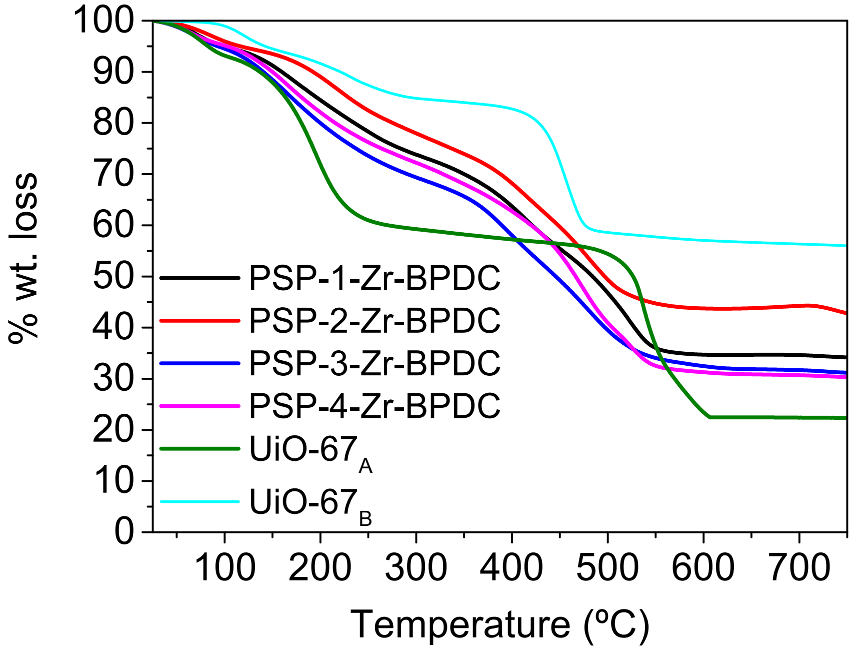

Figure S11. TGA of the PSP-1-4-Zr-BPDC coordination polymers compared with that of UiO-67 (**Zr-BPDC**).

Table S6. TGA analysis (from 25 ºC to 800 ºC (10 ºC/min) under air) of the **PSP-Zr-BPDC** coordination polymers and two samples of UiO-67 with different BPDC content (**Zr-BPDC$_A$** and **Zr-BPDC$_B$**).

**Sample**	**% Guests[a]**	**% organic[b]**	**%ZrO_2[c]**	**Organic/ZrO_2**
**PSP-1-Zr-BPDC**	8	39[d]	35	1,1
**PSP-2-Zr-BPDC**	6	35[d]	43	0,8
**PSP-3-Zr-BPDC**	11	38[d]	32	1,2
**PSP-4-Zr-BPDC**	10	42[d]	31	1,4
**Zr-BPDC$_A$**	12	66	22	3,0
**Zr-BPDC$_B$**	6	38	56	0,7

[a]weight loss below 150 ºC; [b]weight loss (%) between 300 and 600 ºC; [c]weight loss (%) above 600ºC; [d]both PSP and biphenyldicarboxylic acid are included in this mass loss.

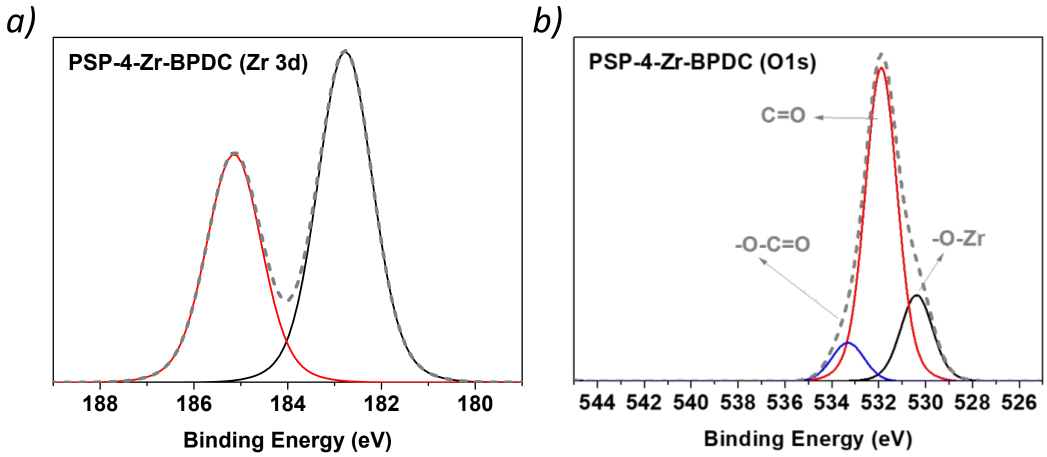

Figure S12. XPS Zr 3d (a) and O1s (b) and signals of the PSP-4-Zr-BPDC coordination polymer.

Table S7. XPS analysis of the PSP-4-Zr-BPDC coordination polymer.

Atomic concentration (%)				Surface Composition
C	N	O	Zr	
63.4	5.3	27.5	3.8	$Zr_1C_{16.7}N_{1.4}O_{7.2}$

Table S8. XPS (O1s) analysis of the PSP-4-Zr-BPDC coordination polymer. Binding energies and percentage of deconvoluted bands with respect to the total oxygen (in parentheses).

**O-Zr (%)**	**C=O (%)**	**-O-C=O (%)**	**-OH (%)**
530.4 (19.6)	531.9 (71.9)	533.3 (8.5)	- (0)

S5. CATALYTIC PERFORMANCE OF THE COORDINATION POLYMERS

Hydrolysis of p-nitrophenyl benzoate: 1.12g of HEPES was dissolved in 95 mL of water and 5 mL of acetonitrile. Next, 5.2 mL of the prepared HEPES buffer (pH =7.5) was added to a glass vial containing 5 mg of Zr-PSP. An aqueous solution of 4.5mg of p-nitrophenyacetate (PNPA) in 50mL of the HEPES solution was prepared, and 575 µL of this solution were added to the reaction vial containing the HEPS solution previously added and the catalyst resulting in an initial [PNPA] = 49.5 µM. To test the catalytic activity of the coordination polymers, aliquots were measured by UV-Vis every 5 minutes for 1 hour.

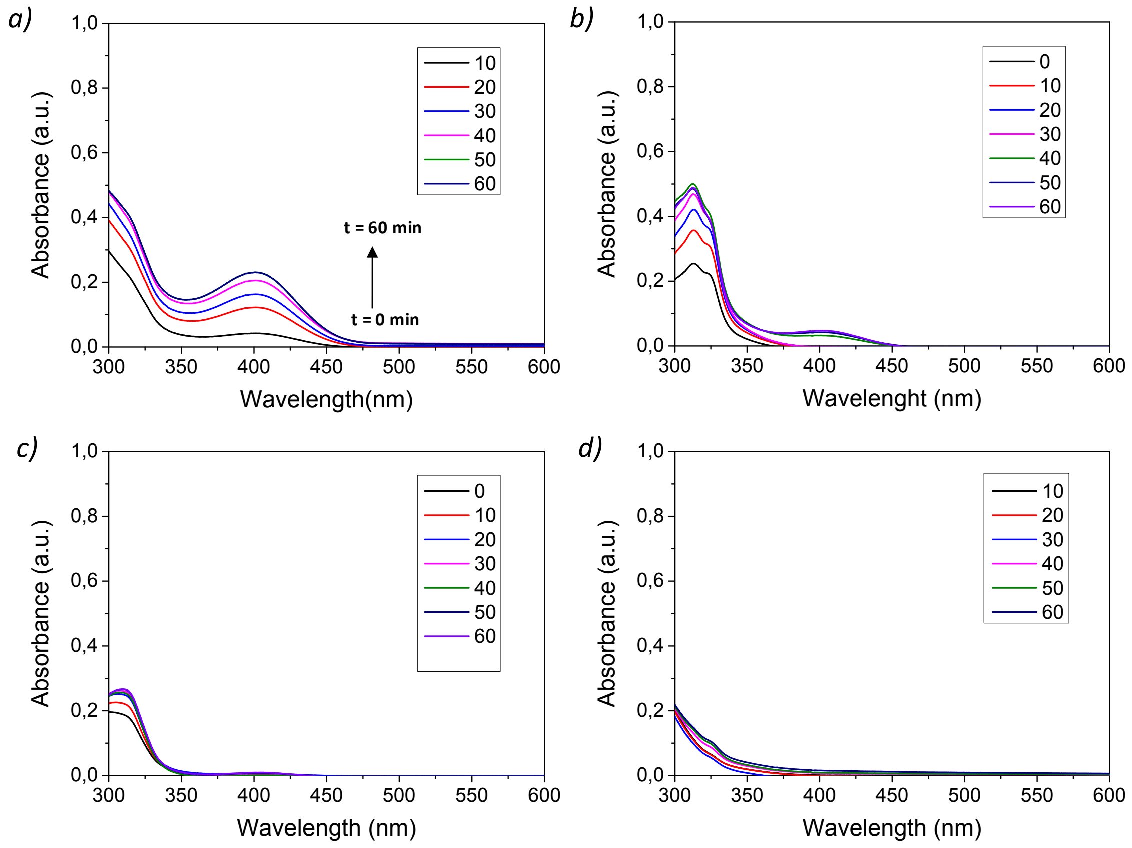

Figure S13. UV-Vis spectrums at different reaction times of the nitrophenol product (λ = 402 nm) from the hydrolysis of p-nitrophenyl benzoate in the presence of **Zr-PSP-1-HFor** (a), **Zr-PSP-1-HAc** (b), **Zr-PSP-1-HCl** (c), $ZrCl_4$ (d).

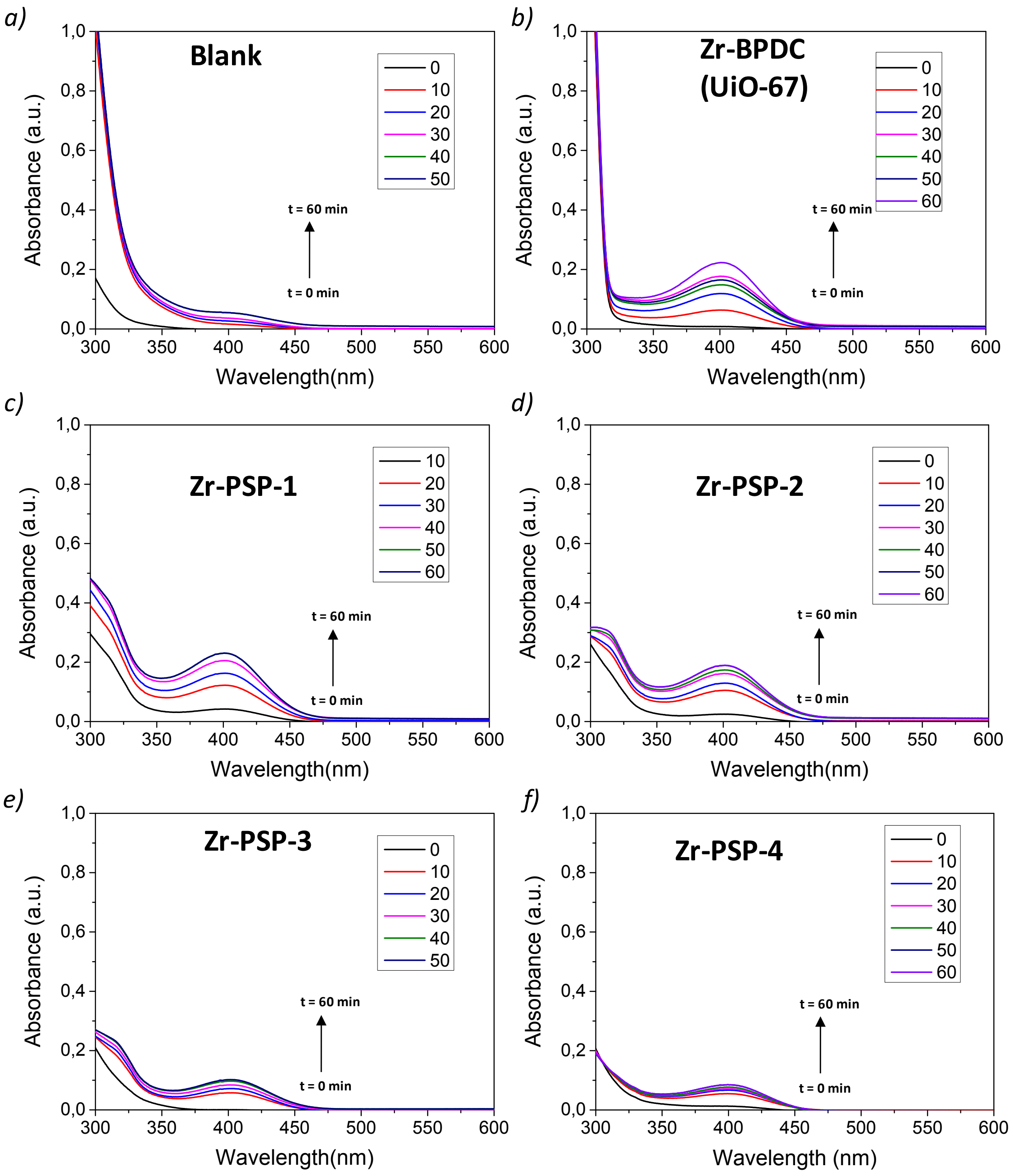

Figure S14. UV-Vis spectra at different reaction times of the nitrophenol product (λ = 402 nm) from the hydrolysis of p-nitrophenyl benzoate in the absence (a) or presence of UiO-67 (b), **Zr-PSP-1** (c), **Zr-PSP-2** (d), **Zr-PSP-3** (e), **Zr-PSP-4** (f).

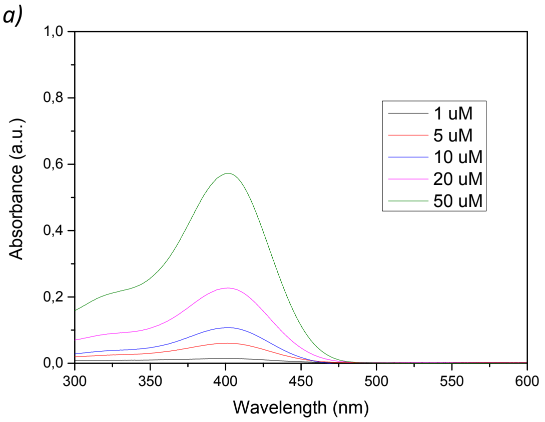

Figure S15. Calibration UV-Vis spectra (a) and resulting fitting with the nitrophenol product concentration (b).

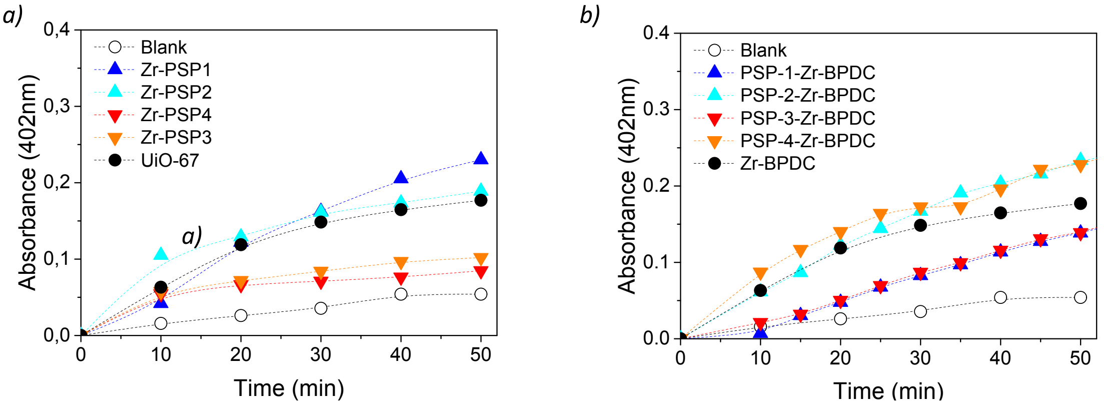

Figure S16. Absorbances obtained from the UV-Vis spectra (and calibration curve) at different reaction times of the nitrophenol product (λ = 402 nm) from the hydrolysis of p-nitrophenyl benzoate in the absence (white) or presence of UiO-67 (black) and the Zr-PSPs (a) or PSP-Zr-BPDC (b). **Zr-PSP-1** or **PSP-1**/UiO-67_{defect} (blue), **Zr-PSP-2** or **PSP-2**/UiO-67_{defect} (cyan), **Zr-PSP-3** or **PSP-3**/UiO-67_{defect} (orange), **Zr-PSP-4** or **PSP-4**/UiO-67_{defect} (red).

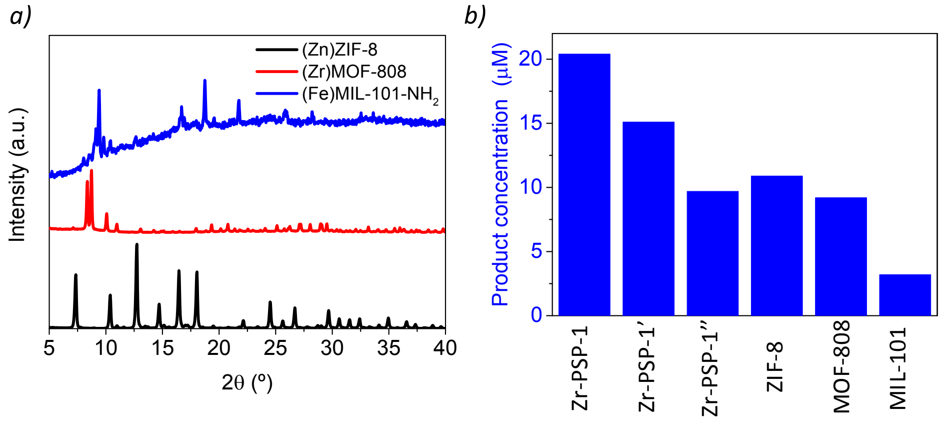

Figure S17. (a) XRD of the MOF tested and (b) product formed after 50 min of hydrolysis of p-nitrophenyl benzoate in the presence of the MOFs. **Zr-PSP-1'** and **Zr-PSP-1''** correspond to the 1st and 2nd use of the **Zr-PSP-1**.

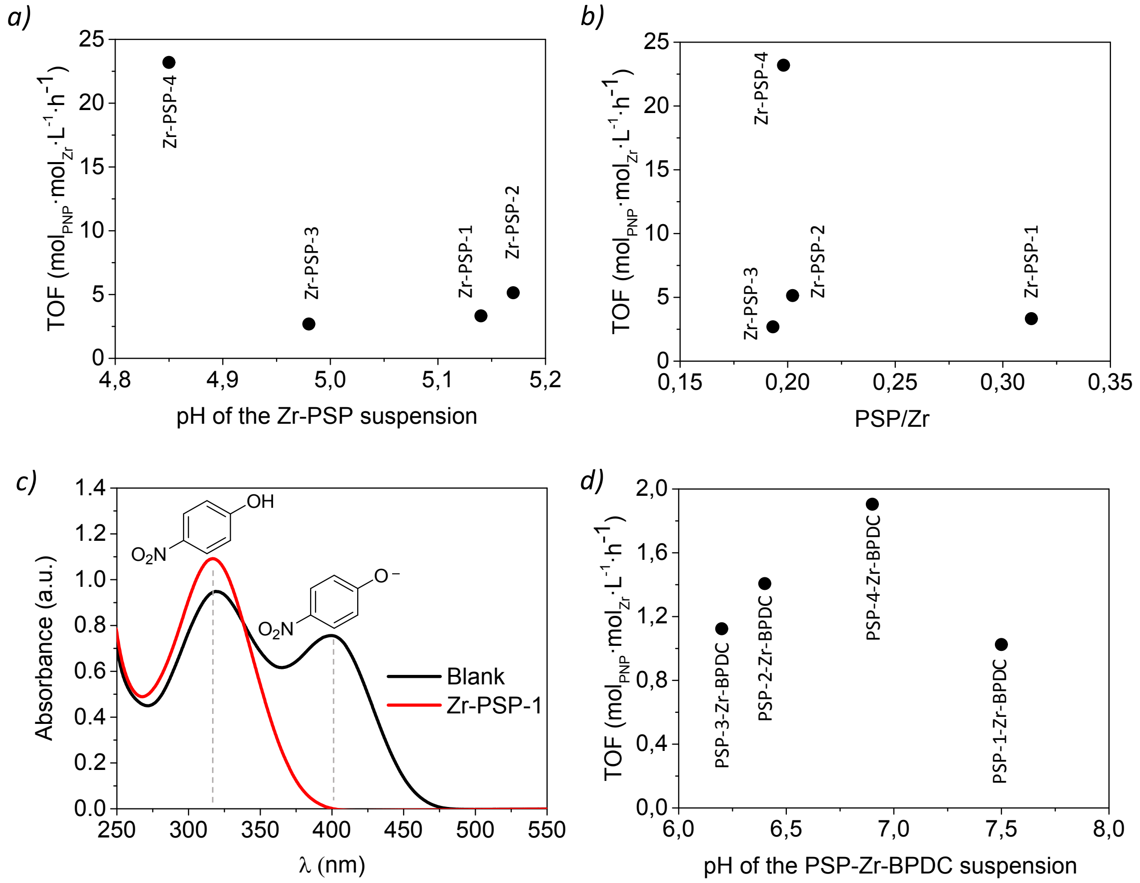

Figure S18. Relationship between catalytic activity and pH of the **Zr-PSP** (1mg/mL) aqueous solution (a and d) or its **PSP**/Zr ratio obtained by TGA (b). UV-Vis spectra of the solution in the presence of **Zr-PSP-1** (c).

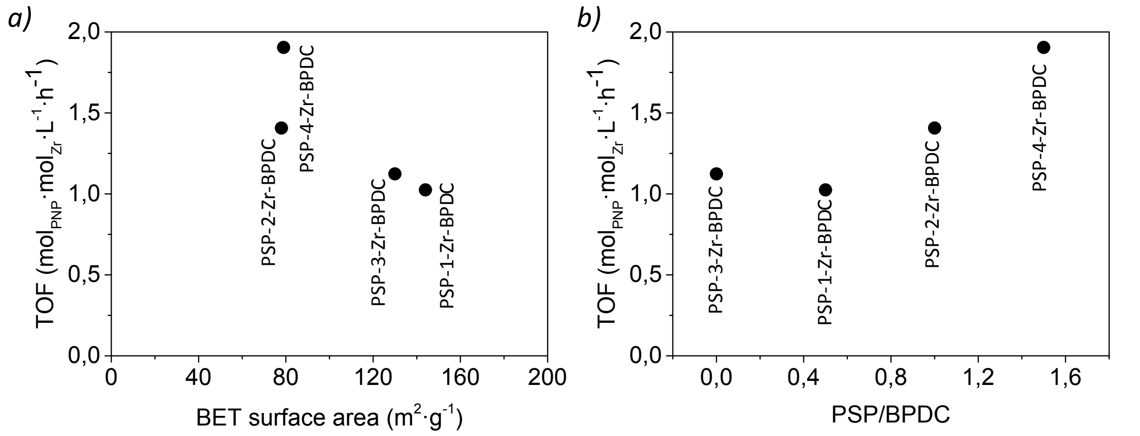

Figure S19. Catalytic performance (expressed either as the TOF of the Zr sites present) of **PSP-Zr-BPDC** samples prepared from the four amino acids at room temperature the hydrolysis of esters with respect to their surface area (a), and the amount of **PSP** incorporated (in moles) with respect to BPDC (b).
